# The Supramolecular Structural Chemistry of Pentafluorosulfanyl and Tetrafluorosulfanylene Compounds

**DOI:** 10.1002/chem.202100163

**Published:** 2021-03-03

**Authors:** Phil Liebing, Cody Ross Pitts, Marc Reimann, Nils Trapp, David Rombach, Dustin Bornemann, Martin Kaupp, Antonio Togni

**Affiliations:** ^1^ Institut für Chemie Otto-von-Guericke-Universität Magdeburg Universitätsplatz 2 39106 Magdeburg Germany; ^2^ Department of Chemistry and Applied Biosciences Swiss Federal Institute of Technology, ETH Zurich Vladimir-Prelog-Weg 2 8093 Zurich Switzerland; ^3^ Institut für Chemie, Theoretische Chemie/ Quantenchemie Technische Universität Berlin Straße des 17. Juni 135 10623 Berlin Germany

**Keywords:** crystal structures, fluorine, halogen bonds, Hammett plot, interaction energy

## Abstract

The analysis of crystal structures of SF_5_‐ or SF_4_‐containing molecules revealed that these groups are often surrounded by hydrogen or other fluorine atoms. Even though fluorine prefers F⋅⋅⋅H over F⋅⋅⋅F contacts, the latter appeared to be important in many compounds. In a significant number of datasets, the closest F⋅⋅⋅F contacts are below 95 % of the van der Waals distance of two F atoms. Moreover, a number of repeating structural motifs formed by contacts between SF_5_ groups was identified, including different supramolecular dimers and infinite chains. Among SF_4_‐containing molecules, the study focused on SF_4_Cl compounds, including the first solid‐state structure analyses of these reactive species. Additionally, electrostatic potential surfaces of a series of Ph‐SF_5_ derivatives were calculated, pointing out the substituent influence on the ability of F⋅⋅⋅X contact formation (X=F or other electronegative atom). Interaction energies were calculated for different dimeric arrangements of Ph‐SF_5_, which were extracted from experimental crystal structure determinations.

## Graphical abstract

**Molecules containing SF_5_ or SF_4_** groups show a diverse solid‐state structural chemistry, including supramolecular dimers or polymers formed by F⋅⋅⋅F or other F⋅⋅⋅X contacts.

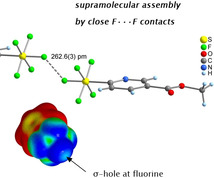

## Introduction

**Figure 1:**
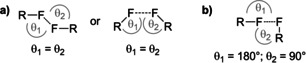
Different arrangements of F⋅⋅⋅F contacts: a) Type I (predominant van‐der‐Waals contacts), b) Type II (“real” halogen bonds according to IUPAC definition^[20]^).

Fluorinated organic compounds are widely used in various fields of application, for example, as pharmaceuticals,^[1]^ crop protectants,^[2]^ and radiomarkers (^18^F PET).^[3]^ Organofluorine chemistry is traditionally focused on fluorine directly bonded to carbon (e.g. in CF_3_, CF_2_, or aryl‐F groups), while functional groups having heteroatom‐bonded fluorine are less investigated. This is certainly due to the challenges associated with synthesizing stable substituents adorned with heteroatom‐fluorine bonds. In the past decade, synthetic strategies have been developed that have made the pentafluorosulfanyl (SF_5_)[^4^, ^5^, ^6^, ^7^, ^8^, ^9^, ^10^] and tetrafluorosulfanylene (SF_4_)[^9^, ^11^, ^12^] groups accessible to a broad chemical community. The SF_5_ moiety, in particular, has attracted attention as a sterically demanding, nonpolar, and electron‐poor group, which is sometimes regarded as “super‐trifluoromethyl group”.^[4]^ Additionally, both SF_5_ and SF_4_ compounds have found interesting applications in materials, such as liquid crystals.[^13^, ^14^] Thus, an in‐depth structural understanding of such compounds seems timely. Organofluorine compounds with carbon‐bound fluorine atoms have been extensively studied regarding their solid‐state structures, which are often governed by attractive F⋅⋅⋅H or F⋅⋅⋅F interactions.[^15^, ^16^] Such interactions are potentially important for the physical properties of materials, as it has been shown with the absorption of fluorinated molecules on solid materials^[17]^ or the gas capture ability of fluorinated metal‐organic frameworks.[^18^, ^19^] Generally, a C−F group is both a poor hydrogen‐bond and halogen‐bond acceptor due to energetically low‐lying fluorine lone‐pairs and low polarizability of fluorine.^[15]^ For these reasons the interactions are relatively weak, but often important for crystal‐structure formation and stabilization. For F⋅⋅⋅F interactions, two different types are described in the literature.^[15]^ “Type I” interactions, which can be described as van der Waals interactions with minimal repulsion contribution, are characterized by similar C−F⋅⋅⋅F angles for both F atoms Figure 1, a). In contrast, “Type II” contacts (which are “real” halogen bonds according to the IUPAC definition^[20]^) are characterized by an l‐shaped structure, having a contact between the nucleophilic region of one F atom and the electrophilic region of the other one (Figure 1, b).

**Figure 2:**
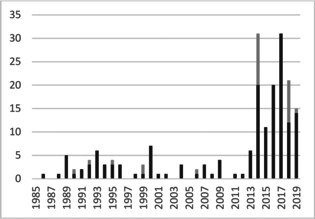
Numbers of SF_5_ (black) and SF_4_ (grey) crystal structures published in the CSD,^[23]^ sorted after publication year (until November 2019).

Except for few crystallographic^[21]^ and computational studies,^[22]^ the supramolecular structural chemistry of SF_5_ and SF_4_ compounds remained largely unnoticed thus far, which motivated us to conduct a detailed study on the solid‐state structural chemistry of these compound classes. In November 2019, the Cambridge Structural Database (CSD)^[23]^ contained no more than 188 entries on compounds of hexavalent sulfur with fluorine substituents (duplicates and SF_6_ solvates excluded), comprising 161 SF_5_ and 27 SF_4_ compounds (see the Supporting Information for details). However, 65 % of all entries have been added during the past six years, attesting to an increasing interest in this young substance class (Figure 2). Similar interaction properties as known for carbon‐bonded fluorine can certainly be expected for sulfur‐fluorine analogues. However, the striking difference between SF_5_ and CF_3_ is the presence of two chemically inequivalent fluorine positions (four equatorial F atoms, F_eq_, and one axial F atom, F_ax_) in the former. Moreover, SF_4_ compounds can exist in two different diastereomeric forms, with the *trans* isomer being usually isolated and structurally characterized for acyclic compounds.[^4^, ^5^, ^6^, ^9^, ^11^, ^12^, ^24^]

## Results and Discussion

Analysis of the SF_5_ crystal structures deposited in the CSD^[23]^ for intermolecular interactions revealed that the SF_5_ group is often surrounded by C−H moieties, thus stabilizing the crystal structures through weak C−H⋅⋅⋅F interactions (Table 1 and Tables S1–S4 in the Supporting Information). The closest F⋅⋅⋅H distance is <260 pm in 56 % of all analyzed structures, being in the usual range of weak F⋅⋅⋅H hydrogen bonds[^16^, ^21^] (for comparison, the sum of van der Waals radii of H and F is 257 pm^[25]^). Contacts to other hydrogen bond donors such as N−H are extremely rare. Generally, the total number of contacts to equatorial F atoms is significantly larger than to the axial one (Table 2). However, it is hard to differentiate if this is simply for statistical reasons since there are four times as many F_eq_ atoms than F_ax_ atoms per SF_5_ group, or if the F_eq_ atoms are actually more potent hydrogen bond acceptors than F_ax_. The majority of F⋅⋅⋅H contacts has been observed in Ar‐SF_5_ compounds (67 %), while F⋅⋅⋅H contacts seem to be less favored in aliphatic R‐SF_5_ compounds (42 %). This finding could be attributed to 1) a higher hydrogen‐bond donor ability of aromatic H atoms as compared to aliphatic ones,^[26]^ and 2) the electronic properties of the additional substituent accompanying the SF_5_ group, thus influencing the hydrogen‐bond acceptor ability of the F atoms.

**Table 1:** General statistics on intermolecular SF_*n*_⋅⋅⋅H and SF_*n*_⋅⋅⋅F contacts in crystal structures of X‐SF_5_ and X‐SF_4_‐Y molecules, including CSD data as well as compounds **1**–**4** reported here (Duplicates, structures with disordered SF_5_ groups, and datasets with *R*_1_ >0.075 omitted).

	Entries total	F⋅⋅⋅H<260 pm^[a]^	279.3 ppm<F⋅⋅⋅F<308.7 pm	F⋅⋅⋅F<279.3 pm
aryl−SF_5_	110	74 (67 %)	62 (56 %)	26 (24 %)
olefinic C(sp_2_)−SF_5_	14	5 (36 %)	7 (50 %)	5 (36 %)
C(sp_3_)−SF_5_	26	11 (42 %)^[b]^	11 (42 %)	6 (23 %)
X−SF_5_ (X=N or O group)	13	4 (31 %)^[b]^	10 (77 %)	–
*trans*‐X‐SF_4_‐Y	25	13 (52 %)	13 (52 %)	1 (4 %)
*cis*‐X‐SF_4_‐Y	4	–	2 (50 %)	1 (25 %)
sum	192	107 (56 %)	105 (55 %)	39 (20 %)

[a] Based on X‐ray crystallographic data and therefore on imprecise determination of hydrogen atomic coordinates. [b] The real abundance might be higher as the H atoms are missing in some datasets.

**Table 2:** Abundance of F_eq_⋅⋅⋅H and F_ax_⋅⋅⋅H contacts among 163 crystallographic datasets with intermolecular X−SF_5_⋅⋅⋅H contacts shorter than 260 pm.

X	Overall entries	Thereof relevant	F_eq_⋅⋅⋅H	F_ax_⋅⋅⋅H
aryl	110	74 (67 %)	48 (44 %)	27 (24 %)
olefinic C(sp_2_)	14	5 (36 %)	4 (29 %)	1 (7 %)
C(sp_3_)	26	11 (42 %)	9 (35 %)	2 (8 %)
N or O group	13	4 (31 %)	3 (23 %)	1 (8 %)
sum	163	94 (58 %)	63 (39 %)	31 (19 %)

Since the hydrogen atom coordinates determined by X‐ray crystallography are not very reliable, we decided to forgo a detailed analysis of F⋅⋅⋅H contact geometries and focus on other F⋅⋅⋅X (X=F, O, N, …) interactions. Even though fluorine prefers F⋅⋅⋅H over F⋅⋅⋅F contacts, the latter turned out to be important in many SF_5_ crystal structures.^[15]^ This is not only the case for molecules not containing hydrogen atoms, but also for many highly functionalized organic molecules. In 20 % of all analyzed SF_5_ crystallographic datasets, the closest F⋅⋅⋅F contacts are below 279 pm, which is <95 % of the van der Waals distance of two F atoms;^[25]^ therefore, these contacts are regarded as significant attractive interactions. In an additional 55 % of SF_5_ datasets, the contacts are in a range of 95–105 % around the van der Waals distance of 294 pm,^[25]^ representing typical van‐der‐Waals contacts. Most of these F⋅⋅⋅F contacts are actually close to Type I geometry according to Figure 1, indicating a high percentage of van‐der‐Waals interaction. However, a number of compounds shows a significant tendency toward Type II behavior, or intermediate cases between both geometries (Tables 3 and S1–S6). Among the Ar‐SF_5_ crystal structures, most of the “strong” F⋅⋅⋅F contacts were observed for molecules where Ar is an electron‐poor or electron‐neutral aryl group, and much less in cases where Ar is a rather electron‐rich aryl group. This finding fits the picture that the halogen bonding ability of fluorine is enhanced by electron‐poor substituents, as it has been discussed earlier.[^15^, ^27^] Actually, most of the observed F⋅⋅⋅F contacts (in both aromatic and aliphatic SF_5_ compounds) stem from equatorial F atoms, which can be expected to be better halogen bond acceptors than F_ax_, as they have another F atom in *trans* position, being more electron‐withdrawing than any other group (Table 4).

**Table 3:** Abundance of Type I and Type II SF_5_⋅⋅⋅F_5_S contacts shorter than 279.3 pm (=95 % of the vdW sum of two F atoms) among 163 crystallographic datasets of X−SF_5_ compounds, according to Figure 1.

X	Overall entries	Thereof relevant	Type I	Type II	Undefined
aryl	110	26 (24 %)	20 (18 %)	3 (3 %)	3 (3 %)
olefinic C(sp_2_)	14	5 (36 %)	3 (21 %)	2 (14 %)	–
C(sp_3_)	26	6 (23 %)	6 (23 %)	–	–
N or O group	13	0 (0 %)	–	–	–
sum	163	37 (23 %)	29 (18 %)	5 (3 %)	3 (2 %)

**Table 4:** Abundance of different SF_5_⋅⋅⋅F contacts shorter than 279.3 pm (=95 % of the vdW sum of two F atoms^[25]^) among 163 crystallographic datasets of X−SF_5_ compounds (F_other_=F atom of substituent other than SF_5_).

	Overall entries	Thereof relevant	F_eq_⋅⋅⋅F_eq_	F_eq_⋅⋅⋅F_ax_	F_eq_⋅⋅⋅F_other_	F_ax_⋅⋅⋅F_ax_	F_ax_⋅⋅⋅F_other_
aryl−SF_5_	110	26 (24 %)	16 (15 %)	6 (5 %)	1 (1 %)	2 (2 %)	1 (1 %)
olefinic C(sp_2_)−SF_5_	14	5 (36 %)	2 (14 %)	1 (7 %)	1 (7 %)	–	1 (7 %)
C(sp_3_)−SF_5_	26	6 (23 %)	4 (15 %)	–	1 (4 %)	1 (4 %)	–
N or O group	13	0 (0 %)	–	–	–	–	–
sum	163	37 (23 %)	22 (13 %)	7 (4 %)	3 (2 %)	3 (2 %)	2 (1 %)

**Figure 3:**
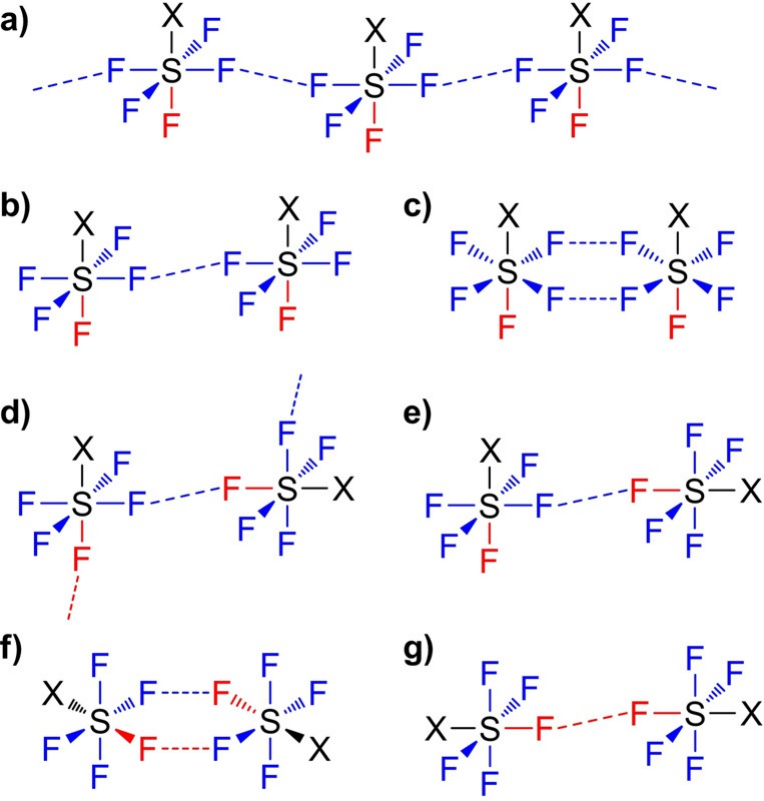
Observed supramolecular structural motifs in crystal structures of SF_5_‐substituted molecules: infinite chains (a, d), open‐chain dimers (b, e, g), and cyclic dimers* (c, f). Blue=F_eq_, Red=F_ax_. *syn or *anti* arrangement of the X groups is possible; shown is the respective *syn* isomer.

Even though F⋅⋅⋅F interactions are expected to be relatively weak and therefore strongly impacted by other intermolecular interactions such as π–π stacking and hydrogen bonds, we identified a number of repeating structural motifs formed by contacts between SF_5_ groups. For more than 50 % of “strong” interactions and also for numerous structures with weak interactions, contacts between equatorial F atoms form supramolecular linear chains (Figure 3 a). Other possible architectures include twisted chains formed by F_eq_⋅⋅⋅F_ax_ contacts (Figure 3 d) and different supramolecular dimers (Figure 3 b, c, e–g).

Analysis of datasets containing other fluorinated groups besides SF_5_ (accounting for ca. 20 % of all datasets) did not allow for a clear conclusion whether SF_5_ does prefer either another SF5 group for F⋅⋅⋅F interactions, or other fluorinated groups. The distribution of “strong” F⋅⋅F interactions between both groups is virtually equal, including SF_5_⋅⋅⋅X contacts with X being an aromatic or aliphatic C‐F, R‐AsF_5_^−^, or PF_6_^−^.

**Figure 4:**
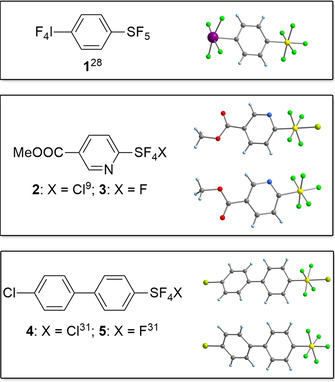
SF_5_‐ and SF_4_Cl‐substituted molecules that have been structurally characterized in the course of this work; note that **2** ^[9]^ and **4** ^[31]^ represent the first examples of SF_4_Cl‐substituted compounds characterized in the solid state.

In the course of our ongoing investigation of perfluorinated main group compounds, we prepared the tetrafluoroiodyl compound F_4_I‐C_6_H_4_‐4‐SF_5_ (**1**, Figure 4; see the Supporting Information for details).^[28]^ In its crystal structure, the molecules are assembled through SF_5_⋅⋅⋅F_5_S and IF_4_⋅⋅⋅F_4_I contacts, while SF_5_⋅⋅⋅F_4_I contacts are not realized. The SF_5_ groups form typical supramolecular chains by F_eq_⋅⋅⋅F_eq_ interactions, but in spite of the very high group electronegativity of the IF_4_ substituent,^[29]^ the interactions are relatively weak with separations of 288.8(3) pm. IF_4_⋅⋅⋅F_4_I contacts arise from direct I⋅⋅⋅F interactions and can therefore be estimated to be stronger than SF_5_⋅⋅⋅F_5_S interactions.^[30]^

The search for F⋅⋅⋅E interactions with E other than F in the CSD disclosed “strong” contacts (<95 % of the corresponding vdW sums) in only a small number of cases, including a diazonium salt (CSD refcode EQACIK; F⋅⋅⋅N 278(1) pm), a sulfonate salt (NALWIG; F⋅⋅⋅O 282.6(3) pm), and a polybrominated porphyrin complex (AGACAP; F⋅⋅⋅Br 301.9(6) pm). Additionally, weak van der Waals contacts between SF_5_ groups and N, O, S, Cl, or B atoms were observed in only 27 % of all cases where these heteroatoms are present, and therefore F⋅⋅⋅F contacts seem to be preferred over other F⋅⋅⋅E contacts. Many of these examples contain very electron‐poor contact groups such as ‐NO_2_, CO ligands, or ‐N_2_^+^, suggesting that fluorine acts more likely as Lewis base rather than as Lewis acid.

The data available for compounds bearing SF_4_ groups did not show clear trends as identified for SF_5_ compounds. This is due to 1) the much lower number of published datasets and 2) the tendency of X‐SF_4_‐Y compounds toward secondary F⋅⋅⋅H and F⋅⋅⋅F bonding, which seems to be lower than for SF_5_ in general. The latter finding might be attributable to steric shielding of the SF_4_ core by the two organic substituents. Moreover, an SF_4_ fragment is always less electron‐deficient than an SF_5_ one, and the F atoms should therefore exhibit a lower tendency toward halogen bonding. Consequently, only 45 % of the analyzed structures contain an F⋅⋅⋅H contact below 260 pm, and only two out of 27 structures (CSD refcodes JOPFID and RESHUV) feature a very close F⋅⋅⋅F contact at 267.6(2) pm. A reasonable comparison between *cis*‐ and *trans*‐SF_4_ derivatives was not feasible since most of the available datasets are *trans*‐SF_4_ compounds.

Among X‐SF_4_‐Y molecules, we were particularly interested in X‐SF_4_Cl compounds, which are important intermediates for the synthesis of X‐SF_5_ as well as other X‐SF_4_‐Y compounds.[^4^, ^5^, ^6^, ^9^, ^11^, ^12^, ^24^] Due to their high reactivity, this compound class resisted structural characterization through X‐ray diffraction thus far. We report here the first two crystal structure analyses of such compounds together with their SF_5_ counterparts (**2**–**5**), allowing for a direct comparison of the supramolecular structural behavior of SF_5_ and SF_4_Cl (see the Supporting Information for details). The S−F_eq_ bond lengths in **2** ^[9]^ (159.2(2)‐161.1(2) pm) and **4** ^[31]^ (158.3(3)‐161.3(3) pm) are slightly longer than in their SF_5_ analogues **3** (155(1)‐160(2) pm) and **5** ^[31]^ (157.5(3)‐160.4(2) pm), respectively. The S−Cl bonds in **2** (206.5(1) pm) and **4** (209.3(2) pm) are within the range observed for other sulfur chlorides in the CSD (ca. 190–210 pm for tetravalent sulfur; values for hexavalent sulfur are not available).^[23]^ The fingerprint plots^[32]^ illustrate that the solid‐state structures of the SF_5_‐ and SF_4_Cl‐substituted molecules are fundamentally different (Figures S13 and S14). The SF_4_Cl group in **2** displays Cl⋅⋅⋅Cl−C and Cl⋅⋅⋅O=C contacts, while in **3** corresponding contacts involving the axial F atom are not present. Instead, compound **3** shows a very close F_ax_⋅⋅⋅F_ax_ contact at 262.6(3) pm. The intermolecular interaction patterns in the 4’‐chlorobiphenyl derivatives **4** and **5** ^[31]^ are more similar than seen with **2** and **3**, displaying a rather close contact of the axial halogen atom to the aryl‐bonded Cl atom (both of Type I geometry), while F⋅⋅⋅F contacts are weak to negligible. The S−Cl⋅⋅⋅Cl−C contact in **4** is 338.8(2) pm (vdW distance: 350 pm^[25]^), and the F_ax_⋅⋅⋅Cl−C contact in **5** measures 313.7(4) pm (vdW distance: 322 pm^[25]^). Additionally, a supramolecular structural similarity for both SF_4_Cl/SF_5_ pairs is that the equatorial F atoms are involved in F⋅⋅⋅H−C hydrogen bonding.

**Figure 5:**
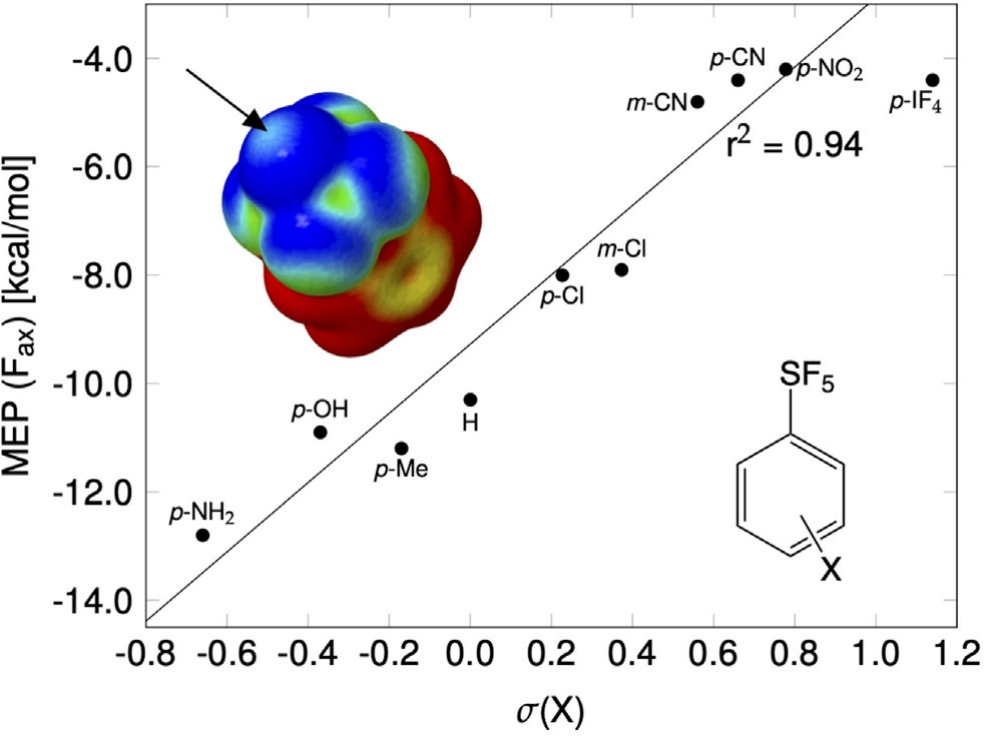
Hammett plot on the σ‐hole at the axial F atom in substituted Ph‐SF_5_ molecules (electrostatic potential surfaces calculated at SCS‐MP2/aug‐cc‐pVTZ level and at a 0.001 electron bohr^−3^ isovalue surface of the electron density). The IF_4_‐substituted molecule (compound **1**) was omitted from the linear regression.

In order to support our crystallographic findings with computational studies, we calculated the electrostatic potential surfaces of a series of simple SF_5_ molecules (see Figure S15 in the Supporting Information). A Hammett plot for a series of simple Ph‐SF_5_ derivatives met the expectation that the magnitude of the σ‐hole at the axial F atom increases with the rising electronegativity of the aryl group (Figure 5). The value of −4.4 kcal mol^−1^, reached for F_5_S‐C_6_H_4_‐4‐CN, seems to be the largest value possible; even the extremely electron‐withdrawing IF_4_ group in **1** does not reinforce the σ‐hole at F_ax_ any more. The substituent influence on the equatorial F atoms was harder to quantify since these atoms do not show a well‐defined σ‐hole. Due to the proximity to the phenyl group and its positive potential, no distinctly localized area of less negative potential was observed. Generally, the trend seems to be similar as for the axial F atom, but significantly weaker. This finding is in agreement with the fact that the F_eq_ atoms have always another F atom in the *trans* position, and the *trans* substituent has a stronger influence on the electronic properties than the *cis* ones.

Finally, we conducted interaction energy calculations for different Ph‐SF_5_ dimers, based on the structures given in Figure 3. These were extracted from real aryl‐SF_5_ crystal structures, with any substituents replaced by hydrogen atoms (see the Supporting Information for details). Due to the increased flexibility of a gas phase dimer compared to the crystal environment, full relaxation of the structures leads to new F−H and F−Ph contacts for all structural motives except for dimers in Figure 3f and g. Therefore, only the phenyl groups were relaxed, keeping the S and F coordinates fixed. Subsequent energy calculations revealed estimated interaction energies between −0.1 and −13.5 kJ mol^−1^, with the largest values for contacts between equatorial F atoms (Table 5). This result fits well with our findings from the crystal structure analyses, where F_eq_⋅⋅⋅F_eq_ appeared most frequently. In order to verify the contribution of non‐F⋅⋅⋅F interactions to the observed interaction energies, we also performed calculations on corresponding acetylene‐SF_5_ systems. The resulting energies are significantly smaller than for the Ph‐SF_5_ dimers, suggesting that the latter are additionally stabilized by F⋅⋅⋅H interactions involving the *ortho*‐C−H moieties close to the SF_5_ groups, or π–π stacking in the case of the *cisoid* F_eq_⋅⋅⋅F_eq_ contact (motif b). However, the single F_eq_⋅⋅⋅F_eq_ and the dual F_eq_⋅⋅⋅F_eq_ contact (Figure 3 b and c, respectively) still show the most negative bonding energies, corroborating the assumption that the contacts between equatorial F atoms are most favorable.

**Table 5:** Interaction energies in kJ mol^−1^ for the R−SF_5_ dimers shown in Figure 3, calculated at SCS‐MP2‐F12 level using a cc‐pVTZ‐F12 basis set and counterpoise corrections in order to account for the basis set superposition error.

Contact	F⋅⋅⋅F [pm]	R=Ph	R=‐C≡CH
F_eq_⋅⋅⋅F_eq_ (b), *anti*	269.3	−10.1	−5.9
F_eq_⋅⋅⋅F_eq_ (b), *syn*	258.8	−9.3	±0.0
dual F_eq_⋅⋅⋅F_eq_ (c)	289.4	−13.5	−8.0
F_eq_⋅⋅⋅F_ax_ (e)	272.1	−4.6	−1.7
dual F_eq_⋅⋅⋅F_ax_ (f)	293.2	−2.1	−2.7
F_ax_⋅⋅⋅F_ax_ (g)	285.8	−0.1	−0.9

## Conclusions

In summary, SF_5_ is a nonpolar, bulky, relatively inflexible group,[^13^, ^22^] whose structural chemistry involves mainly weak secondary bonding interactions. Even though these interactions are strongly impacted by other intermolecular interactions, we could observe some significant trends:

1. The equatorial F atoms show a stronger tendency towards F⋅⋅⋅F contact formation than the axial one; this can be traced back to the strongly electron‐withdrawing fluorine substituent in the *trans* position, while the axial F atom usually has an organic group *trans* to it.
2. F⋅⋅⋅F contacts usually exhibit “Type I” geometry, indicating a large percentage of van‐der‐Waals interaction.
3. Electron‐poor substituents on aryl‐SF_5_ compounds seem to promote F⋅⋅⋅F bond formation; the influence on the axial F atom is thereby larger than on the equatorial ones.
4. F⋅⋅⋅E contacts with E other than F also exist, where E is usually an electron‐poor N or O group.
5. Among the related SF_4_ compounds, SF_4_Cl compounds show the richest supramolecular structural chemistry, since Cl exerts minimal steric shielding of the SF_4_ core and is a better halogen bond acceptor than F.

## Experimental Section

### General information

Unless otherwise stated, all reactions were carried out under strictly anhydrous conditions and Ar or N_2_ atmosphere. All solvents were dried and distilled using standard methods. Trichloroisocyanuric acid was used without prior drying or purification. Spray‐dried KF was always weighed out under N_2_ atmosphere in a glove box. All ^1^H, ^19^F, and ^13^C NMR spectra were acquired on either a 300, 400, or 500 MHz spectrometer. For ^19^F NMR yield determination, trifluorotoluene was introduced after each reaction as an internal standard, and the d1 relaxation delay was increased to 10 s during data collection. The ^1^H, ^13^C, and ^19^F NMR chemical shifts are given in parts per million (ppm) and calibrated to either residual solvent signal (^1^H and ^13^C),^[33]^ α,α,α‐trifluorotoluene (^19^F, *δ*=−63.10 ppm in CD_3_CN),^[34]^ or CFCl_3_ (^19^F, *δ*=−0.65 ppm in CDCl_3_).^[34]^ NMR data are reported in the following format: chemical shift (integration, multiplicity (s=singlet, d=doublet, t=triplet, q=quartet, quintet=quint, m=multiplet), coupling constants (Hz)). IR data were collected on a Thermo Fischer Scientific Nicolet 6700 FT‐IR equipped with a PIKE technologies GladiATR or a PerkinElmer BX II using ATR FT‐IR technology and absorption maxima are reported in cm^−1^. GC/MS was performed on a Thermo Fischer Trace GC 2000 equipped with a flame ionization detector, using a ZB‐5 column with guardian (L: 30 m, i.d.: 0.25 mm, DF=0.25 μm) and helium as the carrier gas with a constant flow of 1.1 mL min^−1^ and a Shimadzu‐QP 2010 Ultra using HP‐5 column with a parallel MS and FID detection. HRMS data were collected by MoBiAS—the MS‐service of the “Laboratorium für Organische Chemie der ETH Zürich”. Single‐crystal X‐ray diffraction data were collected on a XtaLAB Synergy Dualflex Pilatus 300 K Diffractometer, at *T=*100(2) K. Absorption correction was applied on the intensity data using the multi‐scan method.^[35]^

Deposition numbers 2013886 (for **1**), 2013887 (for **2**), 2013888 (for **3**), and 2013889 (for **4**) contain the supplementary crystallographic data for this paper. These data are provided free of charge by the joint Cambridge Crystallographic Data Centre and Fachinformationszentrum Karlsruhe Access Structures service. Crystallographic data and details on structure refinement for the compounds are reported in Table 6.

**Table 6:** Crystal data and details on structure refinement for the compounds **1**–**4**.

Compound	**1**	**2**	**3^[a]^**	**4^[b]^**
CCDC	2013886	2013887	2013888	2013889
molecular formula sum	C_6_H_4_F_9_IS	C_7_H_6_ClF_4_NO_2_S	C_7_H_6_F_5_NO_2_S	C_12_H_8_Cl_2_F_4_S
formula weight [g mol^−1^]	406.05	279.64	263.19	331.14
crystal system	orthorhombic	monoclinic	monoclinic	triclinic
space group	*Pbca*	*P*2_1_/*c*	*P*2_1_/*m*	P$\bar{1}$
*a* [Å]	6.9632(2)	6.1797(1)	6.2352(4)	7.8901(6)
*b* [Å]	8.8136(2)	21.2904(4)	19.7193(16)	8.4624(7)
*c* [Å]	32.914(1)	7.8226(2)	12.2091(11)	10.5314(9)
α [°]	90	90	90	106.565(8)
β [°]	90	105.680(2)	103.599(3)	108.756(7)
γ [°]	90	90	90	94.523(7)
*V* [Å^3^]	2020.0(1)	990.91(4)	1459.1(2)	626.8(1)
molecules per cell *z*	8	4	6	2
electrons per cell *F*_000_	1520	560	792	332
*ρ*_calcd_ [g cm^−3^]	2.670	1.874	1.797	1.754
*μ* [mm^−1^] (radiation)	3.484 (Mo_Kα_)	5.900 (Cu_Kα_)	0.394	6.549 (Cu_Kα_)
crystal shape and color	colorless block	colorless needle	colorless plank	colorless plate
crystal size [mm]	0.08×0.06×0.04	0.22×0.04×0.02	0.28×0.09×0.07	0.22×0.10×0.02
*θ* range [Mo]	3.177 … 34.741	4.153 … 79.748	3.947 … 27.497	4.696 … 79.990
reflns collected	28 728	10776	17079	11743
reflns unique	3982	2116	3375	2629
reflns with *I*>2σ(*I*)	3388	1941	2877	2242
completeness of dataset	99.8 %	100 %	98.3 %	99.7 %
*R*_int_	0.0333	0.0440	0.0418	0.0447
parameters; restraints	154; 0	146; 0	256; 57	172; 6
*R*_1_ (all data, *I*>2σ(*I*))	0.0422; 0.0338	0.0661; 0.0625	0.0464; 0.0382	0.0918; 0.0843
*wR*_2_ (all data, *I*>2σ(*I*))	0.0718; 0.0697	0.1740; 0.1714	0.1093; 0.1058	0.2593; 0.2512
GooF (*F* ^2^)	1.137	1.076	1.144	1.068
max. residual peaks	−1.680; 1.590	−0.911; 0.903	−0.616; 0.339	−1.129; 1.410

[a] Twinned sample; HKLF5 used for final refinement.^[36]^ One molecule is disordered over a mirror plane. This measurement represents the best of many attempts; crystals were always very small and decomposed or redissolved rather quickly, especially after the vessel had been opened. Apart from these problems the refinement quality indicators are reasonable and the structural parameters are very similar to comparable compounds published in the CSD.^[23]^ [b] The moderate data quality is due to the fact that the crystals showed a layered platelet structure. The highest residual peaks hint at a full‐molecule disorder (pseudorotation about 180°), with very low occupancy of the second orientation. It cannot be excluded that the disordered part is a similar but different species. Aryl‐SF_3_, and to some extent, aryl‐SOF_3_ and aryl‐SO_2_F have been repeatedly observed in the product solution, so it is likely one of these compounds has co‐crystallized on the same position. However, none of these could be modelled as a disorder, which is plausible due to the low contribution and partial overlap of atomic positions. This would lead to collisions between adjacent cells, but crystal morphology hints at multicrystallinity or a form of twinning which could simulate disorder. The unmodelled disorder causes ambiguities in the Hirshfeld test for S and Cl, as well as some residual peaks and a relatively high *wR*_2_ value.

### General procedure for the synthesis of aryl tetrafluoro‐λ^6^‐sulfanyl chloride compounds^[9]^

Trichloroisocyanuric acid (0.958 g, 4.1 mmol, 18 equiv) was added to an oven‐dried microwave vial equipped with a stir bar; the vessel was then transported inside a glove box under N_2_ atmosphere. Spray‐dried potassium fluoride (0.425 g, 7.3 mmol, 32 equiv) and the corresponding disulfide (0.23 mmol, 1.0 equiv) were added to the reaction vessel, followed by 4 mL MeCN and trifluoroacetic acid (1.8 μL, 0.02 mmol, 0.1 equiv). The vessel was then sealed with a cap with septum using a crimper, and the reaction mixture was stirred vigorously at room temperature overnight (ca. 18 h). Upon reaction completion, an aliquot of the reaction mixture was passed through a PTFE syringe filter, and an NMR sample was prepared with 0.4 mL of the filtered aliquot and adding 0.1 mL internal standard solution (made immediately prior to use with defined amounts of α,α,α‐trifluorotoluene and CD_3_CN) for ^19^F NMR yield determination.

In order to remove KF and TCICA (and its byproducts), the reaction vessel atmosphere and solvent was purged with Ar and transported into the glove box. Subsequently, the crude reaction mixture was filtered into a PFA vessel via syringe filter and concentrated in vacuo. Then, the crude reaction mixture was diluted with *n*‐hexane, filtered into a PFA vessel, and concentrated in vacuo. (*Note that repeating dilution/filtration/concentration 3–4 times will provide better results due to limited solubility of the aryl‐SF_4_Cl compounds in n‐hexane*.) The crude material consisted of mostly the aryl‐SF_4_Cl product (amount quantified by ^19^F NMR) and was carried forward without further purification.

### General procedure A for the synthesis of pentafluorosulfanyl compounds^[5]^

The aryl‐SF_4_Cl compound (0.12 mmol, 1.0 equiv) was added to a PFA vessel under N_2_ atmosphere in a glove box. Subsequently, AgF (0.36 mmol, 3.0 equiv) was added, and the vessel was sealed and removed from the glovebox. The sealed vessel was heated to 120 °C for ca. 2 days. Upon cooling, the vessel was rinsed with copious amounts of CH_2_Cl_2_ and H_2_O into a separatory funnel. The reaction mixture was extracted with CH_2_Cl_2_. The combined organic layers were dried with MgSO_4_, filtered through Celite, and concentrated. The crude reaction mixture was purified via gradient column chromatography on silica gel on a Teledyne‐Isco Combiflash instrument, eluting with hexanes:EtOAc.

### General procedure B for the synthesis of pentafluorosulfanyl compounds

The aryl‐SF_4_Cl compound (0.046 mmol, 1.0 equiv) was added to a PFA vessel under N_2_ atmosphere in a glove box. Subsequently, AgF (0.287 mmol, 6.2 equiv) was added, and the vessel was sealed and removed from the glovebox. The sealed vessel was placed in a sand bath and heated to 130 °C (at the bottom of the vessel) for 48 h to avoid sublimation of the substrate to the lid. Upon reaction completion, to the reaction mixture 10.0 μL (11.9 mg, 0.0814 mmol) of α,α,α‐trifluorotoluene were added for ^19^F NMR yield determination. The reaction mixture was extracted with *n*‐pentane and *n*‐hexane. The residue was further subjected to column chromatography.

### Procedure for synthesis of pentafluoro(4‐(tetrafluoro‐λ^5^‐ iodanyl)phenyl)‐λ^6^‐sulfane (1)^[28]^

Trichloroisocyanuric acid (0.350 g, 1.5 mmol, 4.0 equiv) was added to an oven‐dried microwave vial equipped with a stir bar; the vessel was then transported inside a glove box under N_2_ atmosphere. Spray‐dried potassium fluoride (0.131 g, 2.3 mmol, 6.0 equiv) and pentafluoro(4‐iodophenyl)‐λ^6^‐sulfane (0.124 g, 0.38 mmol, 1.0 equiv) were added to the reaction vessel, followed by 4 mL MeCN. The vessel was then sealed with a cap with septum using a crimper and removed from the glove box. The reaction mixture was stirred vigorously at 40 °C for ca. 48 h. Upon reaction completion, an aliquot of the reaction mixture was passed through a PTFE syringe filter, and an NMR sample was prepared with 0.4 mL of the filtered aliquot + 0.1 mL internal standard solution (made immediately prior to use with *x* g of either trifluorotoluene or fluorobenzene in *y* mL CD_3_CN) for ^19^F NMR yield determination.

In order to remove KF and TCICA (and its byproducts), the reaction vessel atmosphere and solvent was purged with Ar and transported into the glove box. Subsequently, the crude reaction mixture was filtered into a PFA vessel, washed with dry MeCN, and then concentrated in vacuo. Then, the crude reaction mixture was diluted with *n*‐hexane, filtered into a PFA vessel, and concentrated in vacuo. (*Note that repeating dilution/filtration/concentration 3–4 times will provide better results due to limited solubility of the aryl‐IF_4_ compound in n‐hexane*.) The crude material consisted of mostly the aryl‐IF_4_ product and was carried forward without further purification.

### Characterization data

*Pentafluoro(4‐(tetrafluoro‐λ^5^‐iodanyl)phenyl)‐λ^6^‐sulfane (**1***): The reaction was run according to the procedure outlined above, and the product was formed in 90 % yield by ^19^F NMR analysis. ^19^F NMR (282 MHz, CD_3_CN): *δ*=+80.76 (1F, quint, *J=*149.0 Hz), +61.79 (4F, d, *J=*149.0 Hz), −26.05 (4F, br s). After extracting the product, colorless crystals suitable for single‐crystal X‐ray diffraction were obtained via slow solvent evaporation of a diisopropyl ether solution under inert atmosphere.

*Methyl 6‐(chlorotetrafluoro‐λ^6^‐sulfanyl)nicotinate (**2**)*: The reaction was run according to the general procedure A, and the product was formed in 65 % yield by ^19^F NMR analysis. ^19^F NMR (282 MHz, CD_3_CN): +123.52 (4F, s). After extracting the product, colorless crystals suitable for single‐crystal X‐ray diffraction were obtained via slow solvent evaporation of a 9:1 *n*‐hexane:CH_2_Cl_2_ solution under inert atmosphere.

*Methyl 6‐(pentafluoro‐λ^6^‐sulfanyl)nicotinate (**3**)*: The reaction was run under the conditions of the general procedure B. The residue was further subjected to column chromatography (Alumina–Brockmann grade I, *n*‐pentane to 7:3 (dichloromethane:*n*‐pentane), *R*_f_=0.5 in dichloromethane:*n*‐Pentane (7:3). The product was obtained in 6 % yield by ^19^F‐NMR analysis. ^1^H NMR (400 MHz, Chloroform‐*d*): *δ*=9.18–9.13 (m, 1 H), 8.55–8.48 (m, 2 H), 7.85 (dd, *J=*8.5, 0.8 Hz, 1 H), 4.01 ppm (s, 3 H). ^19^F NMR (376 MHz, Chloroform‐*d*): *δ*=76.66 (quint, *J=*150.3 Hz), 51.98 ppm (d, *J=*150.1 Hz). ^13^C NMR (100 MHz, Chloroform‐*d*) 167.40 (C_q_), 163.60 (C_q_), 149.77 (CH_Ar_), 140.15 (CH_Ar_), 128.85 (C_q_), 121.41 (CH_Ar_), 53.17 (CH_3_). GC/MS (EI): calcd for C_7_H_6_F_5_NO_2_S [M]^+^: 263.0, found 263.0. Crystals suitable for single‐crystal X‐ray diffraction were obtained via extraction of the crude reaction mixture with *n*‐pentane and *n*‐hexane. The solvent was condensed off under atmospheric pressure and −78 °C in a sealed condensation apparatus. Crystals of the product were obtained as low‐melting colorless needles and have been mounted at a temperature of −30 °C.

*Chloro(4′‐chloro‐[1,1′‐biphenyl]‐4‐yl)tetrafluoro‐λ^6^‐sulfane (**4**)*: The reaction was run according to the general procedure A, and the product was formed in 64 % yield by ^19^F NMR analysis. ^19^F NMR (282 MHz, CD_3_CN): +137.13 (4F, s). After extracting the product, colorless crystals suitable for single‐crystal X‐ray diffraction were obtained via slow solvent evaporation of a 9:1 *n*‐hexane:CH_2_Cl_2_ solution under inert atmosphere.

*(4′‐Chloro‐[1,1′‐biphenyl]‐4‐yl)pentafluoro‐λ^6^‐sulfane (**5**)*: The reaction was run according to the general procedure A, and the product was obtained in 77 % isolated yield (29 mg, 0.09 mmol) as a white solid; m.p. 82.8–84.8 °C. ^19^F NMR (471 MHz, CDCl_3_): +84.60 (1F, quint, *J=*150.2 Hz), +63.24 (4F, d, *J=*150.2 Hz); ^1^H NMR (500 MHz, CDCl_3_): 7.83 (2 H, dm, *J=*8.6 Hz), 7.62 (2 H, br d, *J=*8.6 Hz), 7.52 (2 H, dm, *J*=8.6 Hz), 7.45 (2 H, dm, *J*=8.6 Hz); ^13^C{^1^H} NMR (126 MHz, CDCl_3_): 153.1 (quint, *J=*17.5 Hz), 143.3, 137.5, 134.8, 129.3, 128.5, 127.1, 126.6 (quint, *J=*4.6 Hz). $\tilde{\nu }$_max_ (ATR‐IR): 840 cm^−1^ (br), 813 cm^−1^. HRMS (EI): calcd for C_12_H_8_ClF_5_S [M]^+^: 313.9950, found 313.9947. Colorless crystals suitable for single‐crystal X‐ray diffraction were obtained via sublimation.

### Computational studies

All calculations were performed using the TURBOMOLE program package, version 7.3.^[37]^ Structures were optimized at the SCS‐MP2 level of theory using the aug‐cc‐pVTZ basis set^[38]^ for all atoms (aug‐cc‐pVTZ‐PP and the corresponding ECP for I^[39]^) and the corresponding auxiliary basis sets.[^40^, ^41^] SCF was converged to energy changes below 10^−8^ a.u., structures were optimized to a largest Cartesian gradient component below 10^−4^ a.u. For the substituted monomers, the electrostatic potential and the density were calculated at the same level of theory. All electrostatic potential maps show the electrostatic potential projected on a density iso‐surface of 0.001 a.u. in a range from −15 kcal mol^−1^ (blue) to +15 kcal mol^−1^ (red). All pictures were created using the Jmol package.^[42]^ To estimate the interaction energies of the dimer structures roughly, only the phenyl and the acetylene moieties were optimized, the S and F positions were taken from experimental data. For the computation of the interaction energies, we used the obtained fragments without further re‐optimization. The energies were calculated at the SCS‐MP2‐F12 level of theory using cc‐pVTZ‐F12 basis sets^[43]^ and the corresponding auxiliary basis sets.[^44^, ^45^] To correct for basis set superposition errors, the counterpoise correction was applied.

## Conflict of interest

The authors declare no conflict of interest.

## Supporting information

As a service to our authors and readers, this journal provides supporting information supplied by the authors. Such materials are peer reviewed and may be re‐organized for online delivery, but are not copy‐edited or typeset. Technical support issues arising from supporting information (other than missing files) should be addressed to the authors.

Supplementary
