# Supplementary material for: The Supramolecular Structural Chemistry of Pentafluorosulfanyl and Tetrafluorosulfanylene Compounds
Source: Chemistry. 2021 Mar 3;27(19):6086–93. doi: 10.1002/chem.202100163 (PMC8048635; doi:10.1002/chem.202100163)
Supplement: Supplementary file 1 — Supplementary [file CHEM-27-6086-s001.pdf]

Chemistry–A European Journal

Supporting Information

The Supramolecular Structural Chemistry of Pentafluorosulfanyl and Tetrafluorosulfanylene Compounds

Phil Liebing,*[a] Cody Ross Pitts,[b] Marc Reimann,[c] Nils Trapp,[b] David Rombach,[b] Dustin Bornemann,[b] Martin Kaupp,[c] and Antonio Togni[b]

Table of Contents

1. NMR and GC-MS Spectra S1

2. Single-Crystal X-ray Structural Analyses S4

3. Analysis of SF_5- and SF_4-containing Crystal Structures from the CSD S9

4. Computational Studies S27

1. NMR and GC-MS Spectra

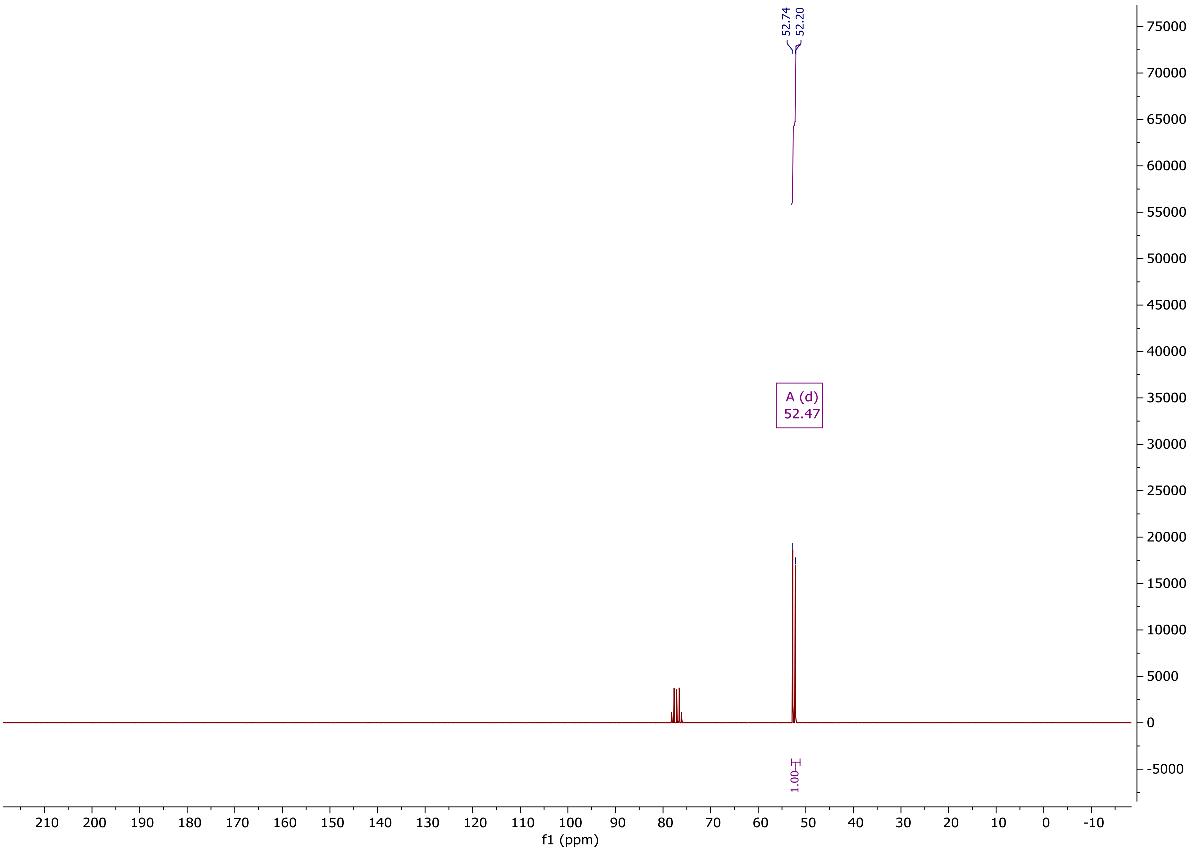

Fig. S1. ^{19}F NMR spectrum of methyl 6-(pentafluoro-λ^6-sulfanyl)nicotinate (**3**).

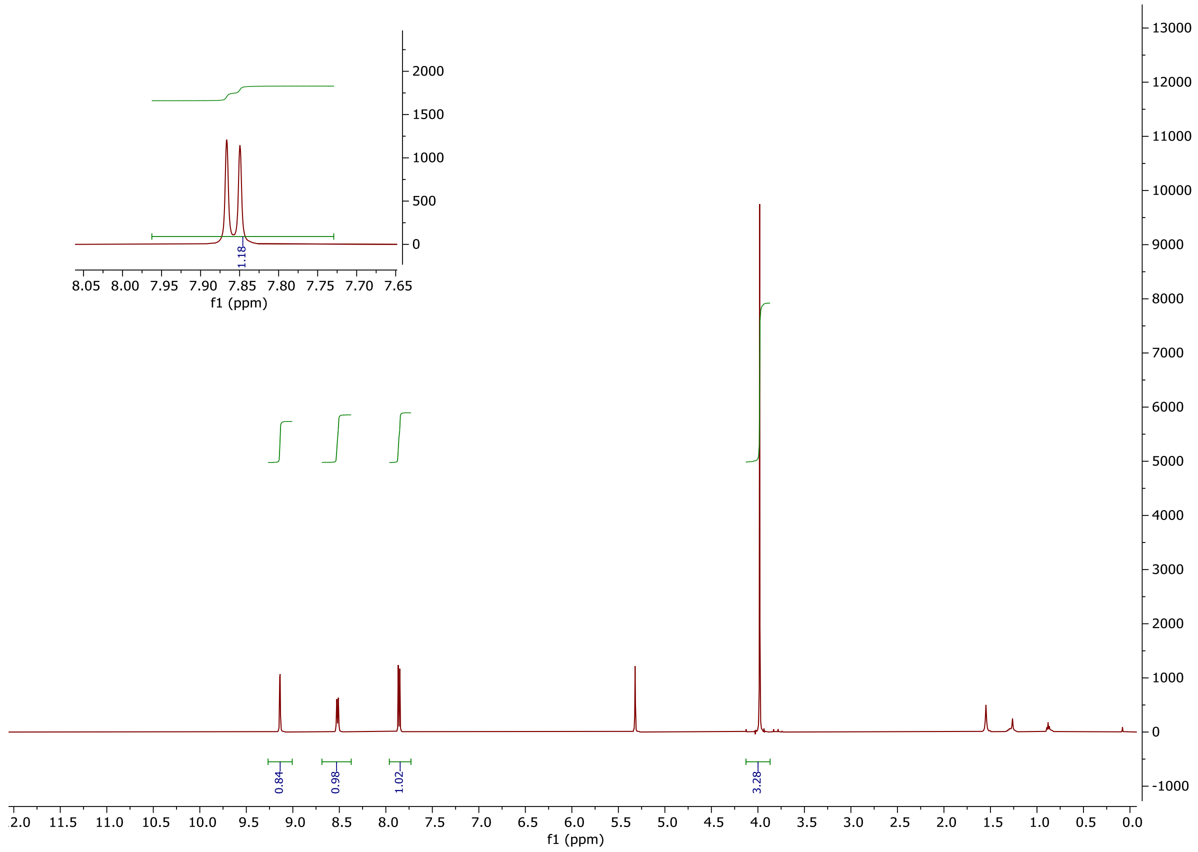

Fig. S2. ^{1}H NMR spectrum of methyl 6-(pentafluoro-λ^{6}-sulfanyl)nicotinate (**3**).

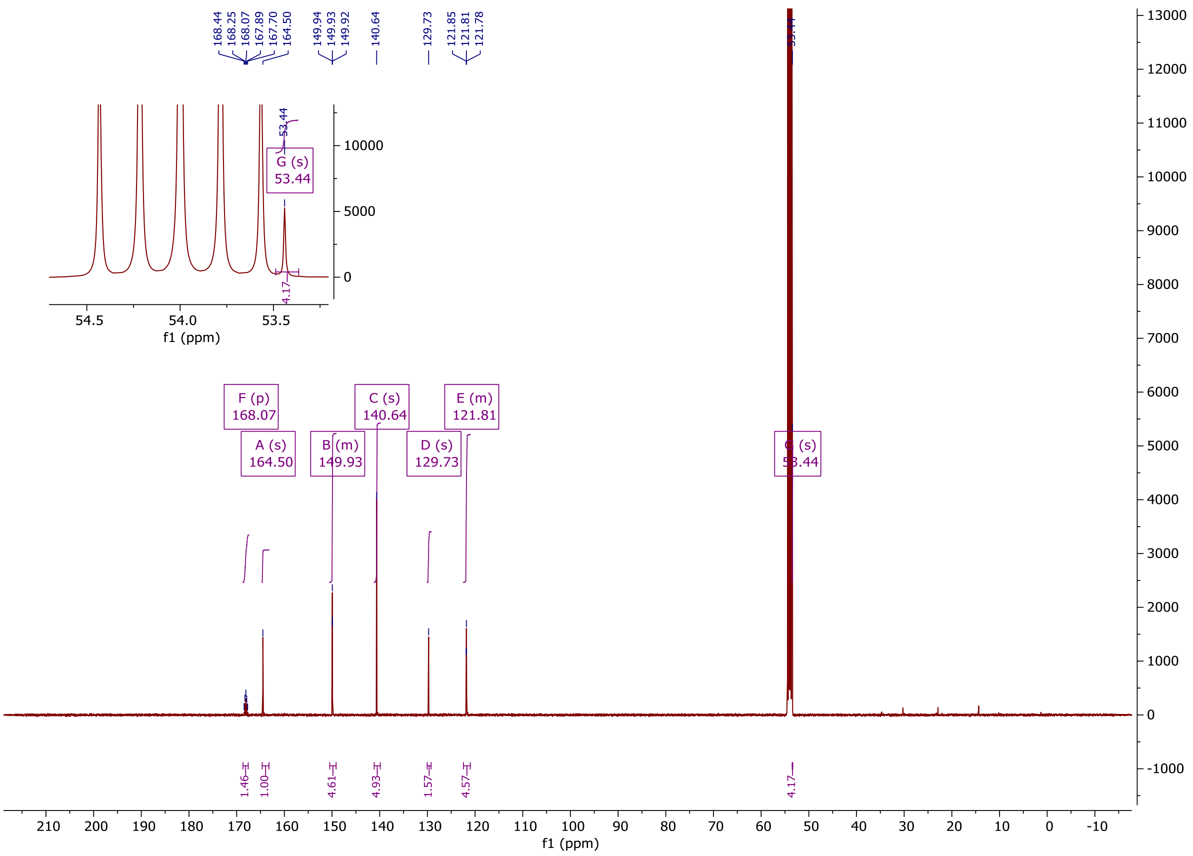

Fig. S3. $^{13}C\{^1H\}$ NMR spectrum of methyl 6-(pentafluoro-λ^6-sulfanyl)nicotinate (**3**).

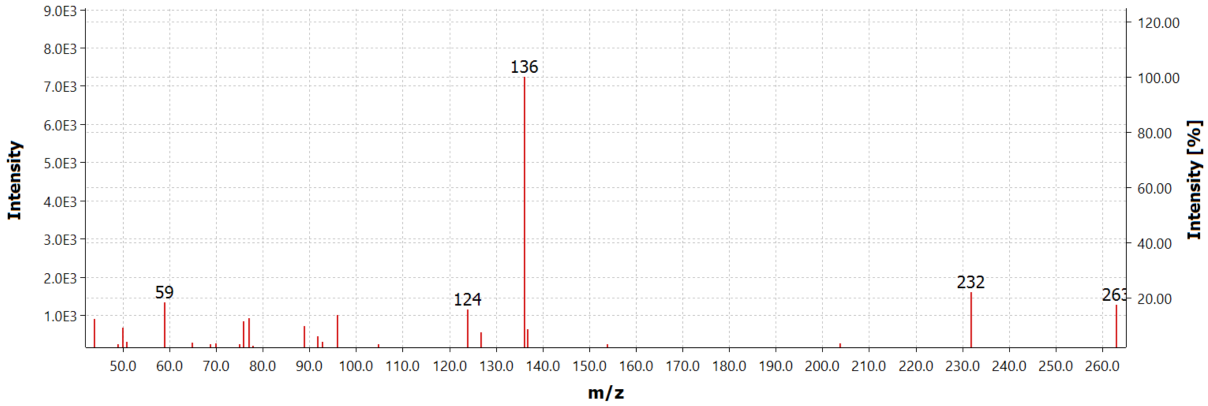

Fig. S4. GC-MS mass spectrum of methyl 6-(pentafluoro-λ^6-sulfanyl)nicotinate (**3**).

2. Single-Crystal X-ray Structural Analyses

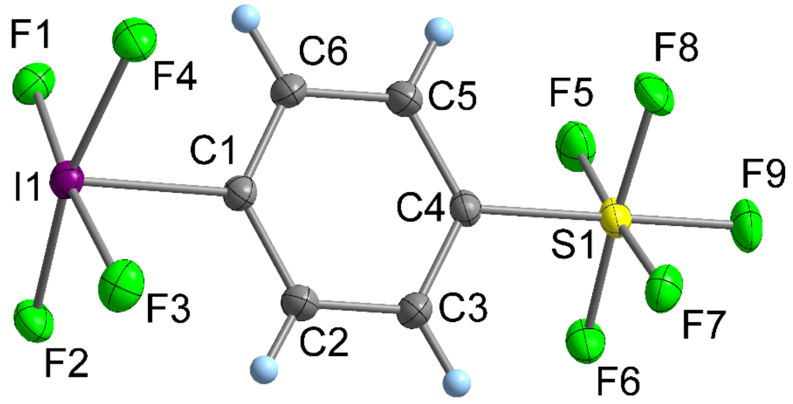

Figure S5. Molecular structure of pentafluoro(4-(tetrafluoro-λ^5-iodanyl)phenyl)-λ^6-sulfane (**1**) in the crystal, showing the atom numbering scheme. Displacement ellipsoids drawn at the 50% probability level.

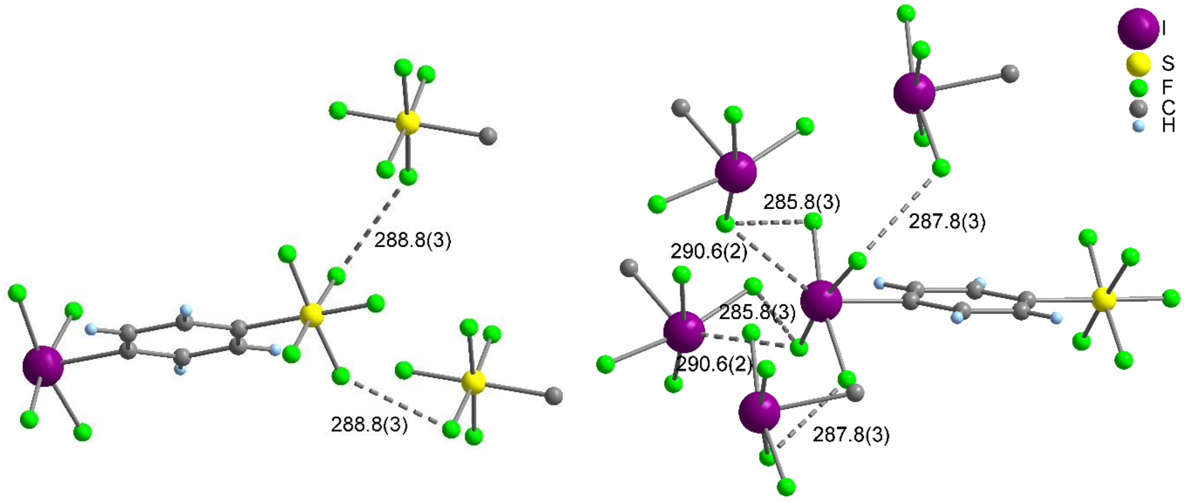

Figure S6. Representation of the $SF_5\cdots F_5S$ and $IF_4\cdots F_4I$ contacts in the crystal structure of **1** (interatomic separations given in pm).

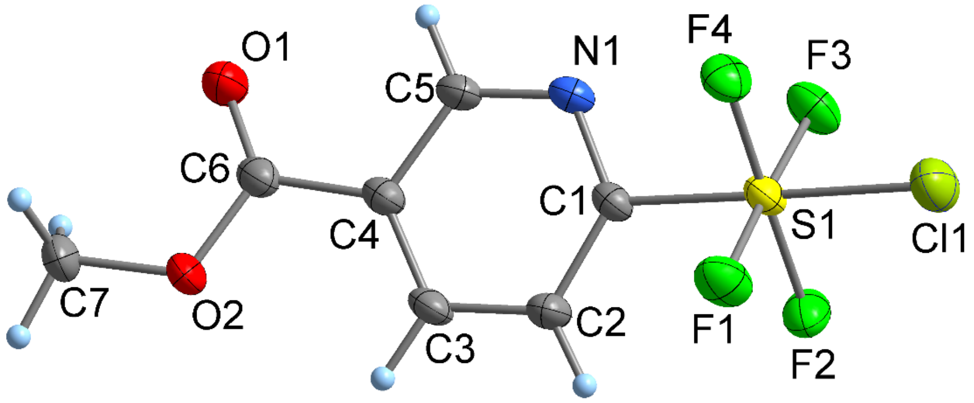

Figure S7. Molecular structure of methyl 6-(chlorotetrafluoro-λ^6-sulfanyl)nicotinate (**2**) in the crystal, showing the atom numbering scheme. Displacement ellipsoids drawn at the 50% probability level.

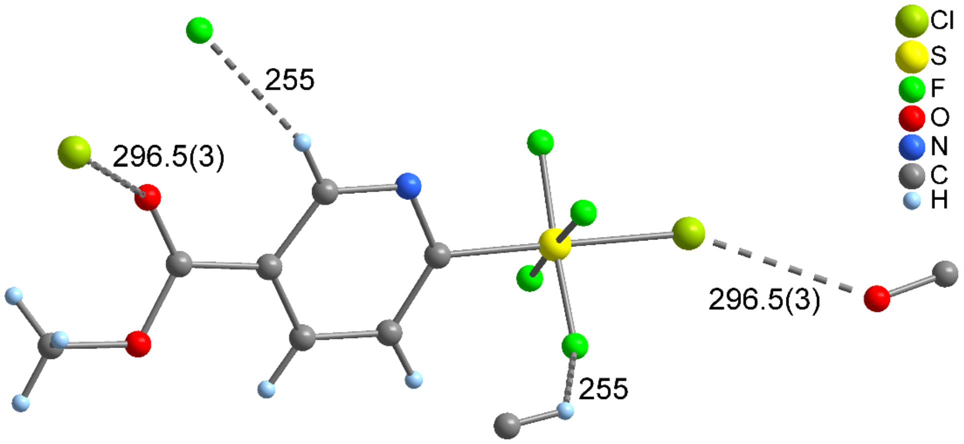

Figure S8. Representation of intermolecular contacts in the crystal structure of **2** (interatomic separations given in pm).

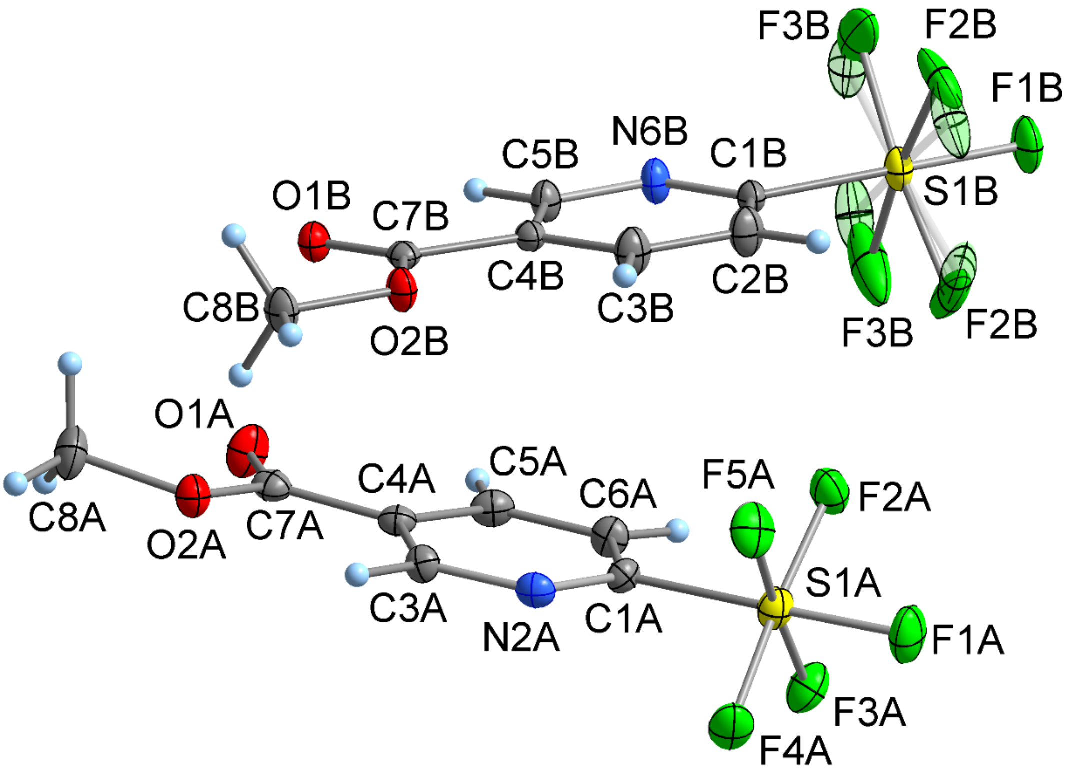

Figure S9. Structures of two symmetry-independent molecules of methyl 6-(pentafluoro-λ^6-sulfanyl)nicotinate (**3**) in the crystal, showing the atom numbering scheme and rotational disorder of the SF_5 group in molecule #2. Displacement ellipsoids drawn at the 50% probability level. Molecule #2 is situated on a crystallographic mirror plane.

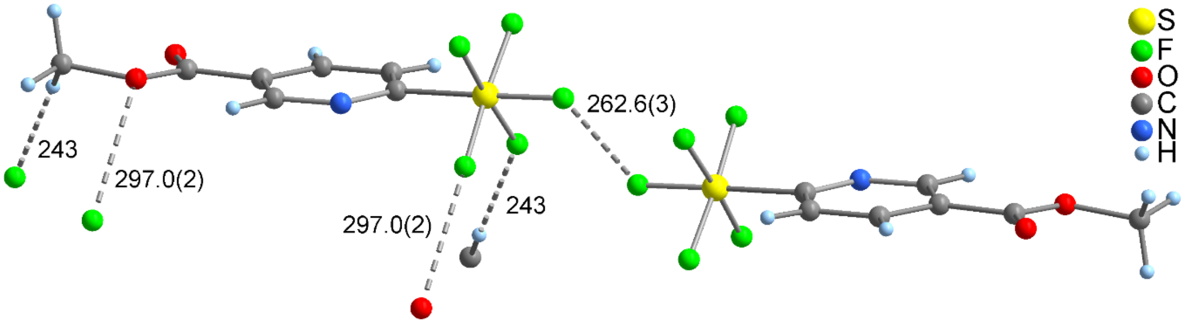

Figure S10. Representation of intermolecular contacts comprising molecule #1 in the crystal structure of **3** (interatomic separations given in pm). All contacts including molecule #2 are significantly longer and therefore not regarded.

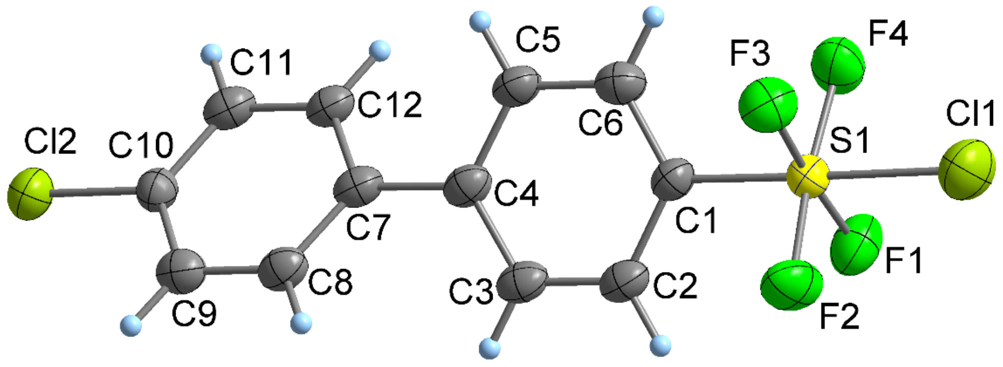

Figure S11. Molecular structure of chloro(4'-chloro-[1,1'-biphenyl]-4-yl)tetrafluoro-λ^6-sulfane (**4**) in the crystal, showing the atom numbering scheme. Displacement ellipsoids drawn at the 50% probability level.

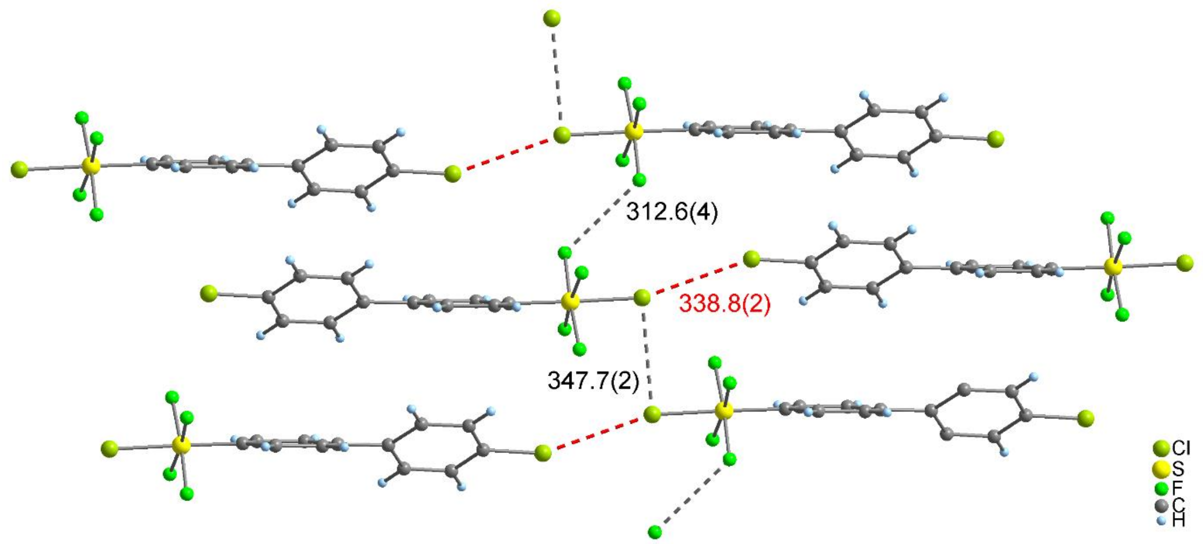

Figure S12. Representation of intermolecular contacts in the crystal structure of **4** (interatomic separations given in pm).

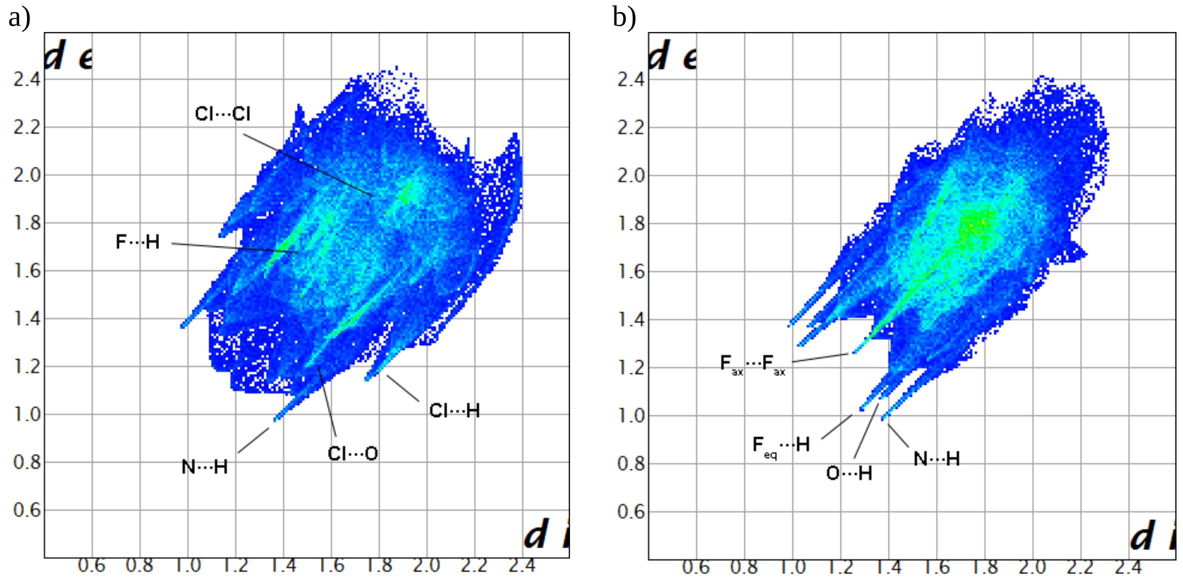

Figure S13. Fingerprint plots[S1] of compounds **2** (a) and the non-disordered molecule #1 in **3** (b), and assignment of the most significant intermolecular interactions. For a general explanation of fingerprint plots, see ref. S2.

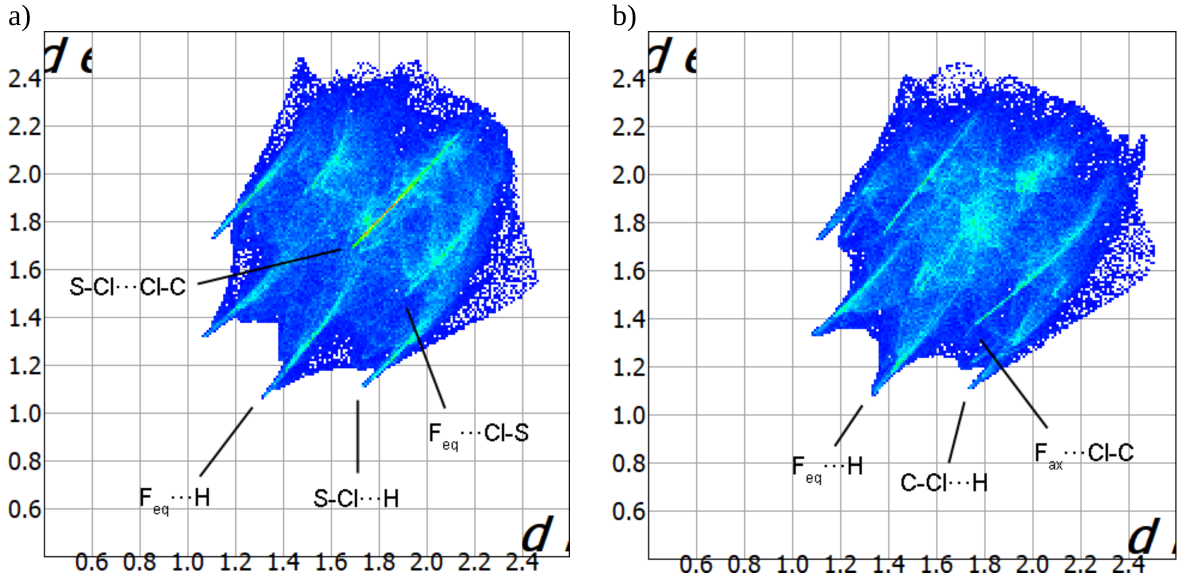

Figure S14. Fingerprint plots [S1] of compounds **4** (a) and **5** (b), and assignment of the most significant intermolecular interactions. For a general explanation of fingerprint plots, see ref. S2.

3. Analysis of SF_5- and SF_4-containing Crystal Structures from the CSD

Color code for Tables S1–S6: Type-I contacts; undefined; Type-II contacts

Table S1. Crystal structures of X-SF_5 compounds where X is an Aryl group (Duplicates, structures with disordered SF_5 groups, and datasets with R_1 > 0.075 omitted).

**CSD Refcode**	**Publ. year**	**X group**	**Closest $SF_5\cdots F_5S$ contact**			**Closest $SF_5\cdots$F(other) contact**			**Closest $SF_5\cdots$E contact**		**Closest $SF_5\cdots$H contact**	
			**Type of contact**	**F···F distance / pm**	**S-F···F angles / deg.**	**Type of contact**	**F···F distance / pm**	**S-F···F-R angles / deg.**	**Type of contact**	**F···F distance / pm**	**Type of contact**	**F···H distance / pm**
LECSAN	1994	(η^5-C_5H_4)Tl(THF)	$F_{eq}\cdots F_{eq}$ chain	281.3(5)	134.6(2), 160.3(2)				–		*(H atom positions questionable)*	
LECSER	1994	(η^5-C_5H_4)Rh(η^4-COD)	$F_{ax}\cdots F_{ax}$ open dimer	303.1(6)	153.8(2), 153.8(2)						$F_{eq}\cdots$H-C	280
ZOVZAL	1996	${}^cCN_2S_2^+$ AsF_6^-	–			$F_{eq}\cdots F_6As^-$	295(2)	144.0(9), 134.3(8)	–			
QAFGOT	1999	C_6H_4-4-${}^cC_4H_6O_2$-4-^{n}Pr	$F_{eq}\cdots F_{ax}$ cyclic dimer	293.2(2)	129.06(8), 136.92(7)				–		$F_{eq}\cdots$H-C	261
OBEJUA	2000	C_6H_4-3-NH-COMe	$F_{eq}\cdots F_{ax}$ chain	294.0(3)	126.4(1), 167.6(1)				–		$F_{ax}\cdots$H-C	272
OBEKAH	2000	C_6H_4-4-NH-COMe	$F_{eq}\cdots F_{eq}$ chain	285.0(3)	121.8(1), 121.8(1)				–		$F_{ax}\cdots$H-C	267
LIPTIO	2007	4-Pyrazolyl	$F_{eq}\cdots F_{eq}$ chain+rings	303.1(4)	116.7(2), 152.8(2)				–		**$F_{eq}\cdots$H-C**	**252**
LIPTOU	2007	4-Triazol-1,2,3-yl	$F_{eq}\cdots F_{eq}$ chain	290.7(2)	115.54(5), 152.46(6),				$F_{eq}\cdots$N	301.7(2)	**$F_{eq}\cdots$H-C**	**255**
TIPMEL	2007	C_6H_4-3-COOH	$F_{eq}\cdots F_{eq}$ chain	308.0(4)	123.0(1), 130.0(1)				$F_{eq}\cdots$O(H)C	304.3(3)	$F_{eq,ax}\cdots$H-C	264
GISVOU	2008	C_6H_4-4-NH_2	$F_{eq}\cdots F_{eq}$ chain	290.3(2)	141.0(1), 144.8(1)				–		**$F_{ax}\cdots$H-N**	**259**
LUGMOQ	2009	bezopyridine derivative	$F_{eq}\cdots F_{eq}$ open dimer	285.6(3)	148.2(1), 148.2(1)	–			–		**$F_{eq}\cdots$H-C**	**254**

CSD Refcode	Publ. year	X group	Closest $SF_5\cdots F_5S$ contact			Closest $SF_5\cdots F$(other) contact			Closest $SF_5\cdots E$ contact		Closest $SF_5\cdots H$ contact	
			**Type of contact**	**F···F distance / pm**	**S-F···F angles / deg.**	**Type of contact**	**F···F distance / pm**	**S-F···F-R angles / deg.**	**Type of contact**	**F···F distance / pm**	**Type of contact**	**F···H distance / pm**
QOVYIK	2009	C_6H_4-4-N(C_6H_3-2-NO_2-4-F)$_2$	–			–			$F_{eq}\cdots O_2N$	290.9(3)	$F_{eq}\cdots H$-C	261
QOVYUW	2009	C_6H_4-4-NO_2	–						$(F_{eq})_2\cdots O_2N$	309.9(2)	$F_{eq}\cdots H$-C	284
AFORAQ	2013	C_6H_4-4-$^{c}C_3H_4F$(COOH)	–			–			$F_{ax}\cdots O$(H)C	306.5(2)	**$F_{ax}\cdots H$-C**	**244**
QIPSAL	2013	naphthyl alcohol	$F_{eq}\cdots F_{eq}$ open dimer	271.8(7)	132.4(3), 154.7(3)						**$F_{eq}\cdots H$-O**	**256**
QIPSEP	2013	benzotetahydrofuran	$F_{eq}\cdots F_{eq}$ chain	292.2(1)	106.41(5), 145.42(6)				–		**$F_{eq}\cdots H$-C**	**259**
DIZROV	2014	C_6H_4-4-CSeNH_2	$F_{eq}\cdots F_{eq}$ chain	288(2)	156.4(4), 160.6(4)				–		$F_{eq}\cdots H$-C	288
DIZRUB	2014	C_6H_4-4-(selenazoline derivative)	–						–		$F_{ax}\cdots H$-C	261
DIZSAI	2014	C_6H_4-4-(selenazoline derivative)	$F_{eq}\cdots F_{ax}$ cyclic dimer	299.2(5)	130.4(2), 141.3(2)				$(F_{eq})_2\cdots O_2N$	297.7(6)	**$F_{eq}\cdots H$-C**	**253**
DIZSEM	2014	C_6H_4-4-(selenazoline derivative)	$F_{eq}\cdots F_{ax}$ cyclic dimer	292.9(6)	129.9(2), 141.6(2)				–		$F_{ax}\cdots H$-C	278
DIZSIQ	2014	C_6H_4-4-(selenazoline derivative)	$F_{eq}\cdots F_{eq}$ chain	280(1)	145.9(4), 148.4(4)				–		**$F_{ax}\cdots H$-C**	**255**
DIZSOW	2014	C_6H_4-4-(selenazoline derivative)	$F_{eq}\cdots F_{ax}$ chain	285.6(7)	114.3(2), 143.8(3)				–		**$F_{eq}\cdots H$-C**	**258**
DIZSUC	2014	C_6H_4-4-(selenazoline derivative)	$F_{eq}\cdots F_{eq}$ chain	305.5(8)	144.6(3), 147.9(3)				–		**$F_{ax}\cdots H$-C**	**257**

**CSD Refcode**	**Publ. year**	**X group**	**Closest $SF_5 \cdots F_5S$ contact**			**Closest $SF_5 \cdots F$(other) contact**			**Closest $SF_5 \cdots E$ contact**		**Closest $SF_5 \cdots H$ contact**	
			**Type of contact**	**F···F distance / pm**	**S-F···F angles / deg.**	**Type of contact**	**F···F distance / pm**	**S-F···F-R angles / deg.**	**Type of contact**	**F···F distance / pm**	**Type of contact**	**F···H distance / pm**
DIZTAJ	2014	C_6H_4-4-(selenazoline derivative)	$F_{eq} \cdots F_{eq}$ cyclic dimer	288.9(4)	117.3(2), 131.9(2)				–		**$F_{ax} \cdots$H-C**	**250**
DIZTEN	2014	C_6H_4-4-$CSNH_2$	**$F_{eq} \cdots F_{eq}$ chain**	**276(1)**	**154.7(4), 158.7(4)**				–		$F_{eq} \cdots$H-C	282
DIZTIR	2014	C_6H_4-4-(N,S-heterocycle)	$F_{eq} \cdots F_{eq}$ chain	300.7(6)	145.8(2), 145.8(2)				–		**$F_{ax} \cdots$H-C**	**254**
DIZTOX	2014	C_6H_4-4-(selenazoline derivative)	$F_{eq} \cdots F_{eq}$ chain	296.5(9)	114.6(3), 132.7(3)						**$F_{eq} \cdots$H-C**	**249**
DIZTUD	2014	C_6H_4-4-(selenazoline derivative)	–								**$F_{ax} \cdots$H-C**	**257**
HOMSOT	2014	$(C_6H_4\text{-4-NH})_2$-CO	$F_{eq} \cdots F_{eq}$ open dimer	269.3(2)	119.88(6), 122.22(6)				–		**$F_{ax} \cdots$H-C**	**246**
HOMSUZ	2014	$(C_6H_4\text{-4-NH-CO-NH})_2\text{-1,2-}C_6H_4$	$F_{eq} \cdots F_{eq}$ open dimer	281.7(4)	134.3(1), 141.4(2)				–		**$F_{ax} \cdots$H-C**	**252**
ZIQJOA	2014	C_6H_4-4-NTf_2	$F_{eq} \cdots F_{eq}$ chain	288.5(9)	138.5(3), 138.5(3)	–			–		$F_{eq} \cdots$H-C	265
ZIQJUG	2014	C_6H_4-4-CH=CH-C_6H_4-4-COOMe	$F_{eq} \cdots F_{eq}$ chain	270.1(1)	145.8(4), 150.1(4)				$F_{eq} \cdots OR_2$	314(1)	$F_{eq} \cdots$H-C	262
ZIQKAN	2014	$(C_6H_4\text{-4-})_2$	$F_{eq} \cdots F_{ax}$ chain	295.5(3)	116.5(1), 151.4(1)				–		**$F_{ax} \cdots$H-C**	**258**
ZIQKER	2014	C_6H_4-4-SCN	$F_{eq} \cdots F_{eq}$ chain	290.1(6)	147.7(3), 151.9(2)				$F_{eq} \cdots$SCN	326.3(5)	$F_{eq} \cdots$H-C	263
EQACEG	2015	C_6H_4-3-N_2^+ BF_4^-	$F_{eq} \cdots F_{ax}$ chain+rings	290.2(5)	110.2(2), 114.8(1)	–			$F_{eq} \cdots N{\equiv}N^+Ar$	291.6(5)	$F_{ax} \cdots$H-C	277
EQACIK	2015	C_6H_4-4-N_2^+ BF_4^-	$F_{eq} \cdots F_{eq}$ open dimer	299.5(8)	116.5(3), 152.2(3)	$F_{eq} \cdots F_4B^-$	285.7(8)	124.8(3), 105.6(5)	**$F_{eq} \cdots N{\equiv}N^+Ar$**	**278(1)**	$F_{eq} \cdots$H-C	270

CSD Refcode	Publ. year	X group	Closest $SF_5\cdots F_5S$ contact			Closest $SF_5\cdots$F(other) contact			Closest $SF_5\cdots$E contact		Closest $SF_5\cdots$H contact	
			**Type of contact**	**F···F distance / pm**	**S-F···F angles / deg.**	**Type of contact**	**F···F distance / pm**	**S-F···F-R angles / deg.**	**Type of contact**	**F···F distance / pm**	**Type of contact**	**F···H distance / pm**
OHOGOJ	2015	C_6H_4-3-IMes$^+$ $CF_3SO_3^-$	$F_{eq}\cdots F_{eq}$ cyclic dimer	289.4(3)	121.3(1), 129.5(1)	$F_{ax}\cdots F_3CSO_2$	282.5(4)	124.5(1), 129.8(4)	–		**$F_{eq}\cdots$H-C**	**258**
UHIBIY	2015	condensed Aryl	–			–					**$F_{ax}\cdots$H-C**	**248**
YUGPUN	2015	C_6H_4-3-Ir$^+$-4-(2-pyridyl) PF_6^-	$F_{eq}\cdots F_{eq}$ open dimer	304.8(6)	124.1(2), 154.4(3)	**$F_{eq}\cdots F_6P^-$**	**269.3(5)**	**174.0(3), 173.6(2)**	–		**$F_{ax}\cdots$H-C**	**245**
YUGQAU	2015	C_6H_4-3-Ir$^+$-4-(pyrazolyl) PF_6^-	$F_{eq}\cdots F_{eq}$ open dimer	291.6(4)	160.8(2), 160.8(2)	–			$F_{eq}\cdots$Cl	329.1(5)	**$F_{ax}\cdots$H-C**	**251**
YUGQEY	2015	C_6H_4-3-(pyrazolyl)-4-Ir$^+$ PF_6^-	$F_{eq}\cdots F_{ax}$ open dimer	285.9(2)	114.1(1), 126.9(1)	–			$F_{ax}\cdots$Cl	330.4(2)	**$F_{eq}\cdots$H-C**	**256**
ARUVAM	2016	C_6H_3-3,5-(boranate)$_2$	$F_{eq}\cdots F_{eq}$ chain	291.9(2)	143.2(1), 143.2(1)				–		$F_{eq}\cdots$H-C	274
ETANEU	2016	C_6H_4-4-CH(OH)-C_6H_4-4-Cl	$F_{eq}\cdots F_{eq}$ chain	282.2(2)	118.30(6), 118.36(6)				–		**$F_{eq}\cdots$H-C**	**252**
ILIHIW	2016	C_6H_3-3-NHPh-4-NO_2	$F_{eq}\cdots F_{eq}$ open dimer	296.1(2)	156.34(7), 156.34(7)				$(F_{eq})_2\cdots O_2N$	303.6(2)	$F_{eq}\cdots$H-C	272
IWUFUD	2016	3-Pyridyl-2-F-4-$NHCH_2Ph$	$F_{ax}\cdots F_{ax}$ open dimer	285.8(2)	162.14(7), 162.14(7)	$F_{eq}\cdots$F-Ar	307.5(1)	141.5(6), 90.7(7)	–		$F_{eq}\cdots$H-C	264
IWUGAK	2016	4-Pyridyl-3-F-5-NH_2	$F_{eq}\cdots F_{ax}$ cyclic dimer	296.5(1)	107.08(5), 132.67(5)	–			$F_{eq}\cdots$N(Py)	314.0(2)	**$F_{eq}\cdots$H-C**	**251**
LABJAC	2016	1,3-C_6H_3-5-PdBr{P(^{i}Pr$_3$}$_2$	$F_{eq}\cdots F_{ax}$ open dimer	286.8(2)	114.80(7), 115.27(8)				–		$F_{eq}\cdots$H-C	269
LABJEG	2016	C_6H_4-3-PdI{P(^{i}Pr$_3$}$_2$	–						–		$F_{eq}\cdots$H-C	261
LABJIK	2016	C_6H_4-4-PdBr{P(^{i}Pr$_3$}$_2$	–						–		**$F_{eq}\cdots$H-C**	**257**
LABJOQ	2016	(C_6H_4-3-)$_2$	$F_{eq}\cdots F_{ax}$ open dimer	297.8(1)	112.36(4), 153.91(5)				–		**$F_{ax}\cdots$H-C**	**255**
LABJUW	2016	1,3-C_6H_3-5-PdF{P(^{i}Pr$_3$}$_2$	$F_{eq}\cdots F_{ax}$ open dimer	286.6(2)	110.00(7), 111.74(6)				–		**$F_{eq}\cdots$H-C**	**254**

**CSD Refcode**	**Publ. year**	**X group**	**Closest $SF_5\cdots F_5S$ contact**			**Closest $SF_5\cdots F$(other) contact**			**Closest $SF_5\cdots E$ contact**		**Closest $SF_5\cdots H$ contact**	
			**Type of contact**	**F···F distance / pm**	**S-F···F angles / deg.**	**Type of contact**	**F···F distance / pm**	**S-F···F-R angles / deg.**	**Type of contact**	**F···F distance / pm**	**Type of contact**	**F···H distance / pm**
LABKAD	2016	$(1,3\text{-}C_6H_3\text{-}5\text{-})_2$	$F_{eq}\cdots F_{eq}$ open dimer	281.4(1)	118.38(5), 161.01(5)				$F_{eq}\cdots B$	293.8(2)	**$F_{eq}\cdots$H-C**	**256**
WAMDIA	2016	C_6H_4-4-IMes$^+$ TosO$^-$	–						–		**$F_{eq}\cdots$H-C**	**242**
BEDHEA01	2017	C_6H_4-4-(alkynyl group)	–						–		**$F_{ax}\cdots$H-C**	**250**
DAPLAK	2017	C_6H_3-3-OH-4-NH_2	$F_{eq}\cdots F_{eq}$ cyclic trimer	279.5(5)	122.2(2), 133.0(2)				–		**$F_{eq}\cdots$H-N**	**243**
DAPLEO	2017	C_6H_4-3-OH	$F_{eq}\cdots F_{eq}$ open dimer	299.1(5)	164.4(1), 164.4(1)				–		**$F_{eq}\cdots$H-C**	**255**
DAPLIS	2017	C_6H_4-4-COOH	**$F_{eq}\cdots F_{ax}$ open dimer**	**272.1(3)**	**131.5(1), 159.9(2)**				$F_{eq}\cdots$O(H)OC	296.8(4)	**$F_{eq}\cdots$H-C**	**257**
DAPLOY	2017	benzopyrazine derivative	$F_{eq}\cdots F_{eq}$ chain	284.6 (5)	110.7(2), 116.4(2)				–		$F_{ax}\cdots$H-C	264
DAPLUE	2017	benzopyrazine derivative	$F_{eq}\cdots F_{eq}$ cyclic dimer	303.5(2)	114.4(1), 155.9(1)				–		$F_{ax}\cdots$H-C	263
DAPMAL	2017	C_6H_3-3-(CHClCOOMe)-4-NO_2	$F_{eq}\cdots F_{eq}$ cyclic dimer	290.8(6)	129.8(2), 136.4(2)				–		–	
DAPMEP	2017	benzoxazoline derivative	**$F_{eq}\cdots F_{ax}$ open dimer**	**279.1(4)**	**118.0(1), 157.6(2)**				–		**$F_{eq}\cdots$H-C**	**255**
DAPMIT	2017	C_6H_3-3-NO_2-4-(CH_2COOEt)	**$F_{eq}\cdots F_{eq}$ chain**	**261.8(9)**	**155.1(3), 157.0(3)**				$F_{eq}\cdots$O=C	318(1)	**$F_{eq}\cdots$H-C**	**258**
DAPMOZ	2017	C_6H_4-4-CN	–						–		**$F_{ax}\cdots$H-C**	**259**
HAPSID	2017	C_6H_3-3-Pt-4-pyridyl	–						–		**$F_{ax}\cdots$H-C**	**256**
HAPSOJ	2017	C_6H_3-3-pyridyl-4-Pt	–						$F_{eq}\cdots$O=C	308.0(3)	$F_{eq}\cdots$H-C	262

**CSD Refcode**	**Publ. year**	**X group**	**Closest $SF_5\cdots F_5S$ contact**			**Closest $SF_5\cdots$F(other) contact**			**Closest $SF_5\cdots$E contact**		**Closest $SF_5\cdots$H contact**	
			**Type of contact**	**F···F distance / pm**	**S-F···F angles / deg.**	**Type of contact**	**F···F distance / pm**	**S-F···F-R angles / deg.**	**Type of contact**	**F···F distance / pm**	**Type of contact**	**F···H distance / pm**
JAYCIY	2017	C_6H_3-3-Ir-4-pyridyl	$F_{eq}\cdots F_{eq}$ cyclic dimer	278.2(4)	107.9(2), 149.3(2)				–		**$F_{eq}\cdots$H-C**	**250**
JAYCOE	2017	C_6H_3-3-pyridyl-4-Ir	–						–		**$F_{eq}\cdots$H-C**	**248**
JAYCUK	2017	C_6H_3-3-Ir-4-pyridyl	$F_{eq}\cdots(F_{eq})_2$ cyclic tetramer	283.7(4)	110.0(1), 169.7(2)				–		**$F_{eq}\cdots$H-C**	**253**
JAYDAR	2017	C_6H_3-3-pyridyl-4-Ir	$F_{ax}\cdots F_{ax}$ open dimer	296.9(7)	124.8(3), 124.8(3)				–		**$F_{eq}\cdots$H-C**	**248**
MEDHUB	2017	C_6H_3-3-Ir-4-pyrazolyl	**$F_{eq}\cdots F_{eq}$ open dimer**	**274.2(4)**	**112.5(2), 147.6(2)**				–		**$F_{eq}\cdots$H-C**	**243**
NANREC	2017	2-pyridyl-4-$IMes^+$ $CF_3SO_3^-$	$F_{eq}\cdots F_{ax}$ open dimer	279.3(3)	127.1(1), 156.7(1)	–			$F_{eq}\cdots O_2S$ (*cation-anion*)	305.0(3)	**$F_{eq}\cdots$H-C**	**242**
RAXWUL	2017	condensed O-heteroaryl	–			–			$F_{eq}\cdots$O=C	286.5(2)	**$F_{eq}\cdots$H-C**	**253**
REFBEM	2017	condensed N-heteroaryl	–						–		**$F_{eq}\cdots$H-C**	**255**
REFBIQ	2017	condensed N-heteroaryl	$F_{eq}\cdots F_{eq}$ chain	275.5(2)	146.9(1), 147.7(1)				–		$F_{eq}\cdots$H-C	271
REFBOW	2017	condensed N-heteroaryl	$F_{eq}\cdots F_{eq}$ cyclic dimer	286.6(2)	111.8(1), 134.3(1)				–		**$F_{eq}\cdots$H-C**	**251**
REFBUC	2017	condensed N-heteroaryl	–						–		**$F_{eq}\cdots$H-C**	**241**
SEFPAX	2017	C_6H_4-4-C_6H_4-4-NPh_2	$F_{eq}\cdots F_{eq}$ open dimer	300.3(3)	127.1(1), 127.1(1)						**$F_{eq}\cdots$H-C**	**252**
SEFPEB	2017	C_6H_4-4-(alkyl group)	–						–		*(organyl disorder)*	

CSD Refcode	Publ. year	X group	Closest $SF_5\cdots F_5S$ contact			Closest $SF_5\cdots F$(other) contact			Closest $SF_5\cdots E$ contact		Closest $SF_5\cdots H$ contact	
			**Type of contact**	**F···F distance / pm**	**S-F···F angles / deg.**	**Type of contact**	**F···F distance / pm**	**S-F···F-R angles / deg.**	**Type of contact**	**F···F distance / pm**	**Type of contact**	**F···H distance / pm**
SEFPIF	2017	C_6H_4-4-(alkynyl group)	–						–		**$F_{eq}\cdots$H-C**	**258**
SEFPOL	2017	C_6H_4-4-(alkynyl group)	–						–		**$F_{eq}\cdots$H-C**	**258**
SEFPUR	2017	C_6H_4-4-(vinyl group)	–						–		*(organyl disorder)*	
SEFWAE	2017	1,3-C_6H_3-5-naphthyl	**$F_{eq}\cdots F_{ax}$ open dimer**	**267(1)**	118.9(5), 152.6(4)	**$F_{eq}\cdots F_3CSO_3^-$**	**257(1)**	141.6(3), 143.6(9)	*(triflyl and Cl^- disorder)*		**$F_{eq/ax}\cdots$H-C**	**246**
XERFIM	2017	$(C_6H_4$-4-$)_3$-pyrazole	**$F_{ax}\cdots F_{ax}$ open dimer**	**277.9(2)**	128.8(1), 155.9(1)				–		**$F_{eq}\cdots$H-C**	**254**
AGACAP	2018	C_6H_4-4-(brominated porphyrine)	**$F_{eq}\cdots F_{eq}$ open dimer**	**277(1)**	115.7(4), 159.9(4)				**$F_{eq}\cdots$Br**	**301.9(6)**	**$F_{ax}\cdots$H-C**	**259**
HIMGAO	2018	C_6H_4-4-NC, Re(I) complex	**$F_{eq}\cdots F_{ax}$ open dimer**	**267.4(6)**	**140.0(3), 147.5(3)**				–		**$F_{ax}\cdots$H-C**	**245**
HIMGES	2018	C_6H_4-4-NC, Re(I) complex	$F_{eq}\cdots F_{ax}$ open dimer	279.8(6)	159.4(2), 164.4(2)	**$F_{ax}\cdots F_5C_6$**	**264.4(5)**	**174.9(2), 160.8(3)**			**$F_{ax}\cdots$H-C**	**255**
JIYFOP	2018	C_6H_4-3-azulenyl	$F_{eq}\cdots F_{eq/ax}$ chain	292.5(3)	121.7(1), 151.5(1)						**$F_{ax}\cdots$H-C**	**247**
PIFBUE	2018	(1,3-C_6H_4-5-NH-CS-$)_2$	**$F_{eq}\cdots F_{eq}$ open dimer**	**266.9(2)**	**143.4(1), 164.3(1)**				–		–	
RERCAV	2018	C_6H_4-4-NC, Cu(I) complex	–			$F_{eq}\cdots F_5C_6$	286.1(1)	169.44(7), 141.34(1)	–		–	
RIJFIC	2018	C_6H_4-4-(alkyl group)	$F_{eq}\cdots F_{eq}$ chain	279.6(4)	133.4(2), 133.7(2)				–		**$F_{eq}\cdots$H-C**	**255**

**CSD Refcode**	**Publ. year**	**X group**	**Closest $SF_5\cdots F_5S$ contact**			**Closest $SF_5\cdots F$(other) contact**			**Closest $SF_5\cdots E$ contact**		**Closest $SF_5\cdots H$ contact**	
			**Type of contact**	**F···F distance / pm**	**S-F···F angles / deg.**	**Type of contact**	**F···F distance / pm**	**S-F···F-R angles / deg.**	**Type of contact**	**F···F distance / pm**	**Type of contact**	**F···H distance / pm**
VEWZUV	2018	1,3-C_6H_3-5-CHO	**$F_{eq}\cdots F_{eq}$ chain**	**277.6(1)**	**139.96(5), 162.86(5)**				–		**$F_{ax}\cdots$H-C**	**236**
WIWJAQ	2018	C_6H_4-2-I^+-Mes TfO^-	**$F_{eq}\cdots F_{ax}$ open dimer**	**275.9(3)**	**141.8(1), 152.0(1)**	$F_{eq}\cdots F_3CSO_2^-$	287.6(4)	113.3(1), 122.3(2)	–		$F_{eq}\cdots$H-C	265
DOJDUE	2019	C_6H_4-4-(N,Se-heterocycle)	$F_{eq}\cdots F_{ax}$ chain	293.0(6)	113.7(2), 168.1(3)				$F_{eq}\cdots O_2N$	290.0(8)	**$F_{eq}\cdots$H-C**	**248**
GEFZIC01	2019	C_6H_4-4-(N-phthalimide)	$F_{eq}\cdots F_{eq}$ chain	258.2(3)	172.6(1), 173.8(1)				–		**$F_{ax}\cdots$H-C**	**249**
HIWZAR	2019	C_6H_4-4-COPh	$F_{eq}\cdots F_{ax}$ chain	288.3(6)	113.0(2), 140.1(2)				–		**$F_{ax}\cdots$H-C**	**242**
LOKXER	2019	(1,3-C_6H_3-5-)$_3$[B-ONa(THF)$_5$]	$F_{eq}\cdots F_{eq}$ open dimer	283.1(2)	135.8(1), 138.5(1)				–		**$F_{eq}\cdots$H-C**	**233**
LOKXIV	2019	[(1,3-C_6H_3-5-)$_4$B]$^-$ Ni(II) salt	**$F_{eq}\cdots F_{eq}$ open dimer**	**267(1)**	**110.6(4), 131.5(4)**				–		**$F_{eq}\cdots$H-C**	**237**
LOPDAY	2019	(C_6H_4-4-O)$_3$P, Ru(II) complex	**$F_{eq}\cdots F_{ax}$ open dimer**	**266(1)**	**138.1(5), 162.3(5)**				$F_{eq}\cdots$O(P)Ar	308(1)	**$F_{eq}\cdots$H-C**	**248**
LOPDUS	2019	(C_6H_4-4-O)$_3$P, Ru(II) complex	**$F_{eq}\cdots F_{eq}$ chain**	291.4(7)	114.1(3), 131.4(3)				–		**$F_{eq}\cdots$H-C**	**255**
ROHSIT	2019	C_6H_4-4-(N,Se heterocycle)	$F_{eq}\cdots F_{eq}$ cyclic dimer	294.8(2)	111.8(1), 141.8(1)				**$F_{ax}\cdots$Se**	**305.7(1)**	**$F_{eq}\cdots$H-C**	**247**
ROHSOZ	2019	C_6H_4-4-(N,Se heterocycle)	–						–		**$F_{ax}\cdots$H-C**	**246**
ROHSUF	2019	C_6H_4-4-(N,Se heterocycle)	$F_{eq}\cdots F_{eq}$ open dimer	300.4(3)	153.2(1), 153.2(1)				$F_{ax}\cdots O_2N$	301.7(3)	**$F_{eq}\cdots$H-C**	**246**

CSD Refcode	Publ. year	X group	Closest $SF_5\cdots F_5S$ contact			Closest $SF_5\cdots$F(other) contact			Closest $SF_5\cdots$E contact		Closest $SF_5\cdots$H contact	
			**Type of contact**	**F···F distance / pm**	**S-F···F angles / deg.**	**Type of contact**	**F···F distance / pm**	**S-F···F-R angles / deg.**	**Type of contact**	**F···F distance / pm**	**Type of contact**	**F···H distance / pm**
ROHTOA	2019	C_6H_4-4-(N-selenourea derivative)	**$F_{eq}\cdots F_{eq}$ cyclic dimer**	**278.2(4)**	**117.8(2), 141.8(2)**						$F_{eq}\cdots$H-C	267
ROHTUG	2019	C_6H_4-4-(N,Se heterocycle)	–						–		**$F_{ax}\cdots$H-C**	**259**
ROHVIW	2019	C_6H_4-4-(N-selenourea derivative)	$F_{eq}\cdots F_{eq}$ cyclic dimer	286(1)	117.9(5), 119.8(5)				–		**$F_{eq}\cdots$H-C**	**246**
TOPMIX	2019	condensed N-heteroaryl	**$F_{eq}\cdots F_{eq}$ open dimer**	**268.2(2)**	**137.8(1), 137.8(1)**						**$F_{eq}\cdots$H-C**	**253**
WOPQOK	2019	C_6H_4-4-C_6H_4-4-Cl	$F_{eq}\cdots F_{ax}$ cyclic dimer	300.8(5)	107.8(1), 128.5(2)				$F_{ax}\cdots$Cl	313.7(4)	**$F_{eq}\cdots$H-C**	**253**
This work		C_6H_4-4-IF_4	$F_{eq}\cdots F_{eq}$ chain	288.8(3)	105.8(1), 144.4(1)	–			–		$F_{eq}\cdots$H-C	260
This work		2-C_5H_4N-4-COOMe	**$F_{ax}\cdots F_{ax}$ open dimer**	**262.6(3)**	**138.5(1), 138.5(1)**				$F_{eq}\cdots OR_2$	297.0(2)	**$F_{eq}\cdots$H-C**	**243**

Table S2. Crystal structures of X-SF_5 compounds where X is a C(sp_2) group other than Aryl (Duplicates, structures with disordered SF_5 groups, and datasets with R_1 > 0.075 omitted).

CSD Refcode	Publ. year	X group	Closest $SF_5\cdots F_5S$ contact			Closest $SF_5\cdots$F(other) contact			Closest $SF_5\cdots$E contact		Closest $SF_5\cdots$H contact	
			**Type of contact**	**F···F distance / pm**	**S-F···F angles / deg.**	**Type of contact**	**F···F distance / pm**	**S-F···F-R angles / deg.**	**Type of contact**	**F···F distance / pm**	**Type of contact**	**F···H distance / pm**
DIVPAZ	1986	C_6H_3-{η^3-$Co(CO)_2$}$_2$	$F_{eq}\cdots F_{ax}$ open dimer	280.1(6)	120.3(2), 125.3(2)				$F_{eq}\cdots O\equiv C$	296.7(9)	**$F_{eq}\cdots$H-C**	**259**
JEBNEJ	1989	(-C=C-){η^2-$Co(CO)_3$}$_2$	$F_{eq}\cdots F_{eq}$ open dimer	303.4(9)	119.9(3), 122.9(3)				$F_{ax}\cdots O\equiv C$	292.0(8)		
KUYYUY	1992	C(=SF_2)CF=N-C_6F_5	–			**$F_{eq}\cdots$F-Ar**	**276.4(4)**	**162.9(2) (S-F), 90.9(3) (C-F)**	$F_{ax}\cdots SF_2$	331.6(3)		
LANWEC	1992	CF=CF-PMe_3^+ BF_4^-	–			**$F_{eq}\cdots$F-C(sp^2)**	**275.4(7)**	**150.8(3), 155.3(4)**	–		$F_{eq}\cdots$H-C	265
LAYHEY	1993	$^-$C(SO_2F)-(COOMe) Et_3NH^+	$F_{eq}\cdots F_{ax}$ chain	303.3(4)	110.6(1), 128.4(2)	–			–		*(H atoms missing)*	
LECRUG	1994	1,4-Norbornadienyl	$F_{eq}\cdots F_{eq}$ open dimer	287.7(2)	160.6(9), 160.6(9)						**$F_{eq}\cdots$H-C**	**258**
ZEZNIB	1995	C=C(CF_3){η^2-($Co(CO)_3$}$_2$	–			$F_{eq}\cdots F_3C$	312(1)	105.0(4) (S-F), 168.6(6) (C-F)	**$F_{eq}\cdots O\equiv C$**	**278.0(1)**		
ZEZNOH	1995	(-CH=CH-CH=CH-)-{η^4-$Co(CO)_2$}-{η^2-$Co(CO)_3$}	**$F_{eq}\cdots F_{ax}$ open dimer**	**273.9(8)**	**126.0(3), 141.5(4)**				$F_{eq}\cdots O\equiv C$	286(1)	*(H atoms missing)*	
ZEZNUN	1995	C=CH{η^2-$Co(CO)_3$}$_2$	$F_{eq}\cdots F_{eq}$ cyclic dimer	302.6(2)	110.4(6), 132.8(8)				$F_{ax}\cdots O\equiv C$	305.6(2)	*(H atoms missing)*	
QEHROL	2006	CH=CH-C_6H_{11}-4-C_6H_{11}-4-^{i}Pr	$F_{eq}\cdots F_{eq}$ chain	283.8(3)	151.2(1), 156.2(1)						$F_{eq}\cdots$H-C	266

CSD Refcode	Publ. year	X group	Closest $SF_5 \cdots F_5S$ contact			Closest $SF_5 \cdots$F(other) contact			Closest $SF_5 \cdots$E contact		Closest $SF_5 \cdots$H contact	
			Type of contact	F···F distance / pm	S-F···F angles / deg.	Type of contact	F···F distance / pm	S-F···F-R angles / deg.	Type of contact	F···F distance / pm	Type of contact	F···H distance / pm
UBAXIG	2011	CH=CH-COPh	**$F_{eq} \cdots F_{eq}$ open dimer**	**272.3(5)**	**128.8(2), 145.3(2)**				$F_{eq} \cdots$O=C	327.7(6)	$F_{eq} \cdots$H-C	269
UTIJUE	2016	^{c}C(=CH)COO-C_4H_4	$F_{eq} \cdots F_{eq}$ open dimer	288.2(1)	111.4(4), 111.4(4)				$F_{eq} \cdots$O=C	288.1(1)	**$F_{eq} \cdots$H-C**	**258**
UTIKAL	2016	C(COOMe)=CH-C_6H_4-4-OMe	–						$F_{eq} \cdots OR_2$	302.0(1)	**$F_{eq} \cdots$H-C**	**257**
ZAGNUT	2016	^{c}C(=CH)CONH-C_4H_4	$F_{eq} \cdots F_{eq}$ open dimer	280.9(3)	121.55(9), 155.20(9)				–		**$F_{ax} \cdots$H-C**	**256**
ZAGPAB	2016	$^{c}C_5H_4$NO	$F_{eq} \cdots F_{eq}$ chain	**276.9(1)**	**124.47(4), 157.80(5)**				$F_{eq} \cdots$O=C	302.9(1)	**$F_{eq} \cdots$H-C**	**256**

Table S3. Crystal structures of X-SF_5 compounds where X is an C(sp_3) group (Duplicates, structures with disordered SF_5 groups, and datasets with $R_1 > 0.075$ omitted).

CSD Refcode	Publ. year	X group	Closest $SF_5 \cdots F_5S$ contact			Closest $SF_5 \cdots$F(other) contact			Closest $SF_5 \cdots$E contact		Closest $SF_5 \cdots$H contact	
			Type of contact	F···F distance / pm	S-F···F angles / deg.	Type of contact	F···F distance / pm	S-F···F-R angles / deg.	Type of contact	F···F distance / pm	Type of contact	F···H distance / pm
JANYAY	1989	(-CH_2)$_3$$^{c}C_3H_3O_3$	$F_{eq} \cdots F_{eq}$ hexameric ring	285.6(6)	129.1(2), 147.6(2)				–		*(H atoms missing)*	
JEBNIN	1989	$CH_2Co(CO)_4$	$F_{eq} \cdots F_{eq}$ chain	302.7(6)	165.3(3), 172.0(3)				$F_{eq} \cdots$O≡C	297.0(9)	–	
JESJUM	1990	CH(CF_2)SO_3	$F_{ax} \cdots F_{ax}$ open dimer	**273.0(7)**	**152.3(3), 152.3(3)**	–			$F_{eq} \cdots O_2S$	296.2(6)	*(H atoms missing)*	
KUYPEZ	1992	CH(SO_2F)-$CONEt_2$	$F_{eq} \cdots F_{ax}$ chain	297.5(6)	110.2(2), 166.4(3)	–			$F_{eq} \cdots$O=C	317.4(5)	$F_{eq} \cdots$H-C	274

**CSD Refcode**	**Publ. year**	**X group**	**Closest $SF_5\cdots F_5S$ contact**			**Closest $SF_5\cdots F$(other) contact**			**Closest $SF_5\cdots E$ contact**		**Closest $SF_5\cdots H$ contact**	
			**Type of contact**	**F···F distance / pm**	**S-F···F angles / deg.**	**Type of contact**	**F···F distance / pm**	**S-F···F-R angles / deg.**	**Type of contact**	**F···F distance / pm**	**Type of contact**	**F···H distance / pm**
YAMGIC	1993	$(-CH_2)_2AsF(OAsF_5)$	$F_{eq}\cdots F_{eq}$ chain	**260.6(6)**	**154.7(3), 166.7(3)**	$F_{eq}\cdots F_5As$	**276.5(7)**	**162.1(3), 173.2(3)**	–		–	
YAMGOI	1993	$CH(CF_3)(AsF_4)$	$F_{eq}\cdots F_{ax}$ chain	305.3(8)	120.7(3), 143.9(3)	$F_{eq}\cdots F_3C$	**272.9(6)**	**162.8(3), 140.8(5)**	–		–	
NALWIG	1996	CH_2-CF_2-SO_3^- $C_{10}H_8S_8^+$	$F_{eq}\cdots F_{eq}$ open dimer	**255.9(3)**	**145.3(1), 145.3(1)**	–			$F_{ax}\cdots {}^-O_3S$	282.6(3)	$F_{eq}\cdots H$-C (cation-anion)	**251**
ITEHIY	1998	$\{-CH(SO_2F)CH_2\}_2O$	$F_{ax}\cdots F_{ax}$ cyclic tetramer	293.7(6)	122.0(2), 150.8(2)	–			–		*(H atoms missing)*	
QATNUU	2000	CH_2-SO_3^- $C_{10}H_8S_8^+$	–						$F_{eq}\cdots S$ (cation-anion)	329.4(4)	$F_{eq}\cdots H$-C (cation-anion)	**243**
QATPAC	2000	CHF-SO_3^- $C_{10}H_8S_8^+$	$F_{ax}\cdots F_{ax}$ cyclic dimer	310.0(4)	114.1(1), 120.2(2)	–			–		$F_{eq}\cdots H$-C	**253**
QATPEG	2000	CF_2-SO_3^- $C_{10}H_8S_8^+$	–			–			$F_{eq}\cdots S$ (cation-anion)	312.9(3)	$F_{eq}\cdots H$-C (cation-anion)	**259**
HUHVUB	2002	CH_2-CH_2-COOH	$F_{eq}\cdots F_{eq}$ chain	311.0(5)	124.2(2), 135.1(2)				$F_{eq}\cdots O$=C	299.2(6)	$F_{eq}\cdots H$-C	274
EYILAZ	2004	CF_2-CF_2-CF_2-CF_2-CH_2-CH_2I	$F_{eq}\cdots F_{eq}$ open dimer	**273.9(8)**	**116.3(4), 142.3(3)**	$F_{eq}\cdots F_2C$	**276.0(8)**	**142.1(5), 164.3(3)**	–		*(undefined)*	
NUSPOI	2004	CF_2-CF_2-C_6H_4-4-N=C-C_6H_4-4-$O^nC_7H_{15}$	$F_{eq}\cdots F_{eq}$ chain	303.8(3)	127.5(1), 128.6(1)	–			–		$F_{ax}\cdots H$-C	277
MOMHUT	2013	CH_2-${}^cC_3H_2NO$-$(OCH_2Ph)(C_6H_4$-4-OMe)	$F_{eq}\cdots F_{ax}$ cyclic dimer	288.3(1)	113.06(4), 136.48(5)				$F_{eq}\cdots OR_2$	311.5(1)	$F_{eq}\cdots H$-C	261

**CSD Refcode**	**Publ. year**	**X group**	**Closest $SF_5\cdots F_5S$ contact**			**Closest $SF_5\cdots F$(other) contact**			**Closest $SF_5\cdots E$ contact**		**Closest $SF_5\cdots H$ contact**	
			**Type of contact**	**F···F distance / pm**	**S-F···F angles / deg.**	**Type of contact**	**F···F distance / pm**	**S-F···F-R angles / deg.**	**Type of contact**	**F···F distance / pm**	**Type of contact**	**F···H distance / pm**
MOMJAB	2013	CHEt-$^{c}C_3H_2NO$-(OCH_2Ph)(C_6H_4-4-OMe)	–						–		$F_{ax}\cdots$H-C	260
LOVROF	2015	$^{c}C_4H_6N$(CH_2Ph)-(CONH-CH_2Ph)	$F_{eq}\cdots F_{eq}$ chain	301.2(5)	131.2(2), 152.4(2)				–		$F_{ax}\cdots$H-C	272
ZUNJEZ	2015	$^{c}C_3H_3NO$(COOEt)-(CH_2Ph)(CO-$^{c}C^{4}H^{8}NO$)	$F_{eq}\cdots F_{eq}$ chain	325.3(3)	115.1(1), 141.4(1)				$F_{eq}\cdots$O=C	313.0(3)	$F_{eq}\cdots$H-C	263
UTIJOY	2016	CH(COOMe)-CH(OH)-C_6H_4-4-NO_2	$F_{eq}\cdots F_{eq}$ chain	**269.1(2)**	**128.19(5), 149.29(6)**				–		**$F_{eq}\cdots$H-C**	**251**
VAKMOM	2016	CH(CHPhOH)-C(O)-OCH_2Ph	$F_{eq}\cdots F_{eq}$ cyclic dimer	313.9(5)	126.9(2), 141.6(2)				$F_{eq}\cdots$O(H)R	315.6(5)	**$F_{ax}\cdots$H-C**	**258**
VAKMUS	2016	CH{CH(OH)-C_6H_3-2,6-Cl_2}-COO-$^{n}C_8H_{17}$	$F_{ax}\cdots F_{ax}$ open dimer	326.8(6)	107.3(2), 107.7(2)				$F_{eq}\cdots$Cl-Ar	328.2(3)	*(alkyl disorder)*	
ZAGNON	2016	CH($COOCH_2Ph$)-CH(OH)-C_6H_4-2-NO_2 · PhF	$F_{eq}\cdots F_{eq}$ cyclic dimer	298.2(2)	120.0(6), 149.6(7)	–			$F_{ax}\cdots$O=C	314.9(2)	**$F_{ax}\cdots$H-C**	**257**
HEZPIO	2018	$^{c}C_4H_5N$(2-naph-thyl)(COOMe)-(COO-CH_2Ph)	–						–		**$F_{eq}\cdots$H-C**	**253**
KEVHIF	2018	CH_2-$^{c}C_3H_2NO$-(OCH_2Ph)(CH_2-CH=CH_2)	$F_{eq}\cdots F_{eq}$ chain	308.4(2)	114.92(6), 141.65(6)				–		**$F_{eq}\cdots$H-C**	**259**
YIQPOG	2018	CH_2-$^{c}C_3H_3NO$-Ph	$F_{eq}\cdots F_{eq}$ open dimer	290.6(3)	135.1(1), 135.1(1)				–		**$F_{eq}\cdots$H-C**	**259**

Table S4. Crystal structures of X-SF_5 compounds where X is a nitrogen or oxygen group (Duplicates, structures with disordered SF_5 groups, and datasets with R_1 > 0.075 omitted).

**CSD Refcode**	**Publ. year**	**X group**	**Closest $SF_5 \cdots F_5S$ contact**			**Closest $SF_5 \cdots$F(other) contact**			**Closest $SF_5 \cdots$E contact**		**Closest $SF_5 \cdots$H contact**	
			**Type of contact**	**F···F distance / pm**	**S-F···F angles / deg.**	**Type of contact**	**F···F distance / pm**	**S-F···F-R angles / deg.**	**Type of contact**	**F···F distance / pm**	**Type of contact**	**F···H distance / pm**
JEBNOT	1989	N=CFS-Mn$(CO)_5$	–			–			$F_{eq} \cdots O \equiv C$	305.7(5)	–	
VAJKUM	1989	$S(NMe_2)_3^+$ SOF_5^-	$F_{eq} \cdots F_{eq}$ chain	327.8(7)	161.8(3), 159.8(3)				$F_{eq} \cdots S^+$	350.4(7)	**$F_{eq} \cdots$H-C**	**259**
JIDPIV	1991	{-NH(CO)O-CH_2}$_2$-C$(NO_2)_2$	$F_{eq} \cdots F_{eq}$ open dimer	285.5(5)	113.8(2), 115.5(2)	–			$F_{eq} \cdots O_2N$	288.6(5)	**$F_{ax} \cdots$H-C**	**259**
JIDPOB	1991	NH(CO)-OCH_2-CF$(NO_2)_2$	$F_{eq} \cdots F_{eq}$ chain	285.0(8)	124.0(3), 141.2(3)				$F_{eq} \cdots OR_2$	296.7(8)	–	
YAVJAG	1993	N=C{O-CH_2-CF$(NO_2)_2$}-{N(NO_2)-CH_2-CH_2-O-NO_2}	$F_{eq} \cdots F_{eq}$ chain	293.3(4)	150.0(2), 152.1(2)				$F_{eq} \cdots O_2N$	292.6(8)	*(H atoms missing)*	
YAVJEK	1993	N=$^cC_3N_2H_2$-$(NO_2)_2$	$(F_{eq})_2 \cdots F_{ax}$ cyclic tetramer	288.2(4)	116.7(2), 116.0(2)				$F_{eq} \cdots O_2N$	299.2(6)	*(H atoms missing)*	
YAVJOU	1993	N=$^cC_3N_2H_3$-(NO_2)	$F_{eq} \cdots F_{eq}$ open dimer	286.5(3)	157.0(1), 157.9(1)				–		*(H atoms missing)*	
NALHUD	1996	N=C{N=C-Cr$(CO)_5$}$_2$	$F_{eq} \cdots F_{eq}$ cyclic dimer	312.7(8)	128.2(3), 135.0(3)				$F_{ax} \cdots O \equiv C$	288.2(8)	–	
KIFHIQ	2000	N=NO_2^- $CN_3H_6^+$	$F_{eq} \cdots F_{eq}$ open dimer	284.2(4)	143.8(1), 143.8(1)				–		**$F_{eq} \cdots$H-N**	**245**
KIFHOW	2000	N=NO_2^- $CN_4H_7^+$	$F_{eq} \cdots F_{eq}$ cyclic dimer + chain	298.9(4)	111.6(1), 129.0(2)				–		**$F_{eq} \cdots$H-N**	**247**
NABZAS	2004	N=CCl_2	$F_{eq} \cdots F_{ax}$ cyclic dimer	307.1(2)	120.1(1), 151.1(1)				–			

CSD Refcode	Publ. year	X group	Closest $SF_5\cdots F_5S$ contact			Closest $SF_5\cdots$F(other) contact			Closest $SF_5\cdots$E contact		Closest $SF_5\cdots$H contact	
			**Type of contact**	**F···F distance / pm**	**S-F···F angles / deg.**	**Type of contact**	**F···F distance / pm**	**S-F···F-R angles / deg.**	**Type of contact**	**F···F distance / pm**	**Type of contact**	**F···H distance / pm**
JUMLIO	2014	O-C_6H_4-4-C_6H_4-4-C_6H_{10}-4-$^nC_3H_7$	$F_{eq}\cdots F_{eq}$ open dimer	297.1(1)	112.4(1), 112.4(1)				–		$F_{ax}\cdots$H-C	265
VAXSIZ	2017	Nitride	$F_{eq}\cdots(F_{ax})_2$ network	302.1(8)	147.6(1), 170.9(3)							

Table S5. Crystal structures of *trans*-X-SF_4-Y compounds (Duplicates and datasets with $R_1 > 0.075$ omitted).

CSD Refcode	Publ. year	X group	Y group	Closest $SF_4\cdots F_4S$ contact		Closest $SF_4\cdots$F(other) contact		Closest $SF_4\cdots$E contact / pm	Closest $SF_4\cdots$H contact / pm
				**F···F distance / pm**	**S-F···F angles / deg.**	**F···F distance / pm**	**S-F···F-R angles / deg.**		
LEZQEM	1999	C_6H_4-4-NO_2	C_6H_4-4-NO_2	–				305.5(4) (O_2N)	266 (H-C)
ECIKAD	2006	C_6H_4-4-NO_2	CF_3	284.4(4)	124.0(2), 124.0(2)			–	270 (H-C)
VIYVUW	2014	Phenyl	CH=CClPh	293.5(1)	117.02(5), 137.00(5)			–	261 (H-C)
VIYWAD	2014	C_6H_4-4-Cl	CH=CClPh	288.8(1)	117.87(4), 138.90(4)			–	273 (H-C)
VIYWEH	2014	C_6H_4-4-NO_2	CH=CClPh	289.7(2)	123.98(7), 123.98(7)			–	261 (H-C)
VIYWIL	2014	C_6H_4-4-Br	CH=CClPh	–				338.0(2) (Br)	**254 (H-C)**
VIYWOR	2014	C_6H_4-4-Ph	CH=CClPh	–				326.1(2) (Cl)	269 (H-C)
VIYWUX	2014	Phenyl	C≡CPh	289.7(3)	110.63(9), 110.63(9)			–	**258 (H-C)**
VIYXAE	2014	C_6H_4-4-Cl	C≡CPh	284.8(1)	114.39(4), 114.39(4)			326.2(1) (Cl)	269 (H-C)

**CSD Refcode**	**Publ. year**	**X group**	**Y group**	**Closest SF_4···F_4S contact**		**Closest SF_4···F(other) contact**		**Closest SF_4···E contact / pm**	**Closest SF_4···H contact / pm**
				**F···F distance / pm**	**S-F···F angles / deg.**	**F···F distance / pm**	**S-F···F-R angles / deg.**		
VIYXEI	2014	C_6H_4-4-NO_2	C≡CPh	289.5(2)	145.7(1), 146.6(1)			–	**259 (H-C)**
ZUCVAW	2014	C_6H_4-4-NO_2	CH=CCl-^{n}Bu	285.0(1)	117.49(4), 125.91(4)			309.8(1) (O_2N)	**252 (H-C)**
ZUCVEA	2014	C_6H_4-4-NO_2	CH_2-CHCl-^{n}Bu	280.4(7)	141.3(3), 141.6(3)			–	**247 (H-C)**
ZUCVIE	2014	C_6H_4-4-NO_2	C≡C-^{n}Bu	–				302.2(2) (O_2N)	**251 (H-C)**
GEJKIS	2018	4-Br-2-Pyridyl	CH-CClPh	–				317.4(1) (Cl)	**254 (H-C)**
RESHUV	2018	CF_3	CH_2-COOH	**267.6(2)**	**148.88(9), 148.88(9)**	287.8(2) (CF_3)	159.2(1) (S-F), 137.3(2) (C-F)	–	**251 (H-C)**
RESJAD	2018	CF_3	CH_2-COPh	282.4(2)	138.64(6), 138.64(6)	*(CF_3 disorder)*		–	**248 (H-C)**
RESJEH	2018	CF_3	CHBr-COPh	291.4(2)	118.02(8), 134.26(8)	*(CF_3 disorder)*		–	263 (H-C)
RESJIL	2018	CF_3	CHCl-C_6H_4-4-Ph	–		*(CF_3 disorder)*			*(alkyl disorder)*
RESJOR	2018	CF_3	CH=CH-COOH	–		*(CF_3 disorder)*		302.1(2) (OH)	–
RESJUX	2018	CF_3	CH_2-CHN=NH-$C_6H_3(NO_2)_2$	292.8(2)	117.26(9), 153.15(9)	290.9(3) (CF_3)	129.6(1) (S-F), 110.1(2) (C-F)	294.1(3) (O_2N)	268 (H-C)
WIHHON	2018	2-C_5H_3N-4-Br	Triazolyl(Aryl)$_2$	281.3(2)	124.43(7), 124.43(7)			321.6(1) (Br)	260 (H-C)
WIHHUT	2018	2-C_5H_3N-4-Br	Triazolyl(Aryl)$_2$	–				323.6(1) (Br)	**253 (H-C)**
HIWZEV	2019	5-COOMe-2-Pyridyl	CH2-CHCl-CH2-CH2Ph	–				–	**241 (H-C)**
(This work)		2-C_4H_3N-4-COOMe	Cl	–				–	**255 (H-C)**
(This work)		C_6H_4-4-C_6H_4-4-Cl	Cl	312.6(4)				–	**251 (H-C)**

Table S6. Crystal structures of *cis*-X-SF_4-Y compounds (Duplicates and datasets with R_1 > 0.075 omitted).

**CSD Refcode**	**Publ. year**	**X group**	**Y group**	**Closest $SF_4\cdots F_4S$ contact**		**Closest $SF_4\cdots$F(other) contact**		**Closest $SF_4\cdots$E contact / pm**	**Closest $SF_4\cdots$H contact / pm**
				**F⋯F distance / pm**	**S-F⋯F angles / deg.**	**F⋯F distance / pm**	**S-F⋯F-R angles / deg.**		
KICDOP	1990	-CH=C(Ph)-O- (ring)		292(1)	115.5(5), 115.5(5)			–	–
JOPFID	1992	-C(CFCl-O-SO_2F)-SO_2-O- (ring)		–	–	**278.8(3)**	**170.6(1) (SF_4), 144.9(1) (SO_3F)**	312.8(2) (Cl), 289.5(3) (O)	
TUNWEE	1995	-CH_2-SO_2-O- (ring)		291.3(3)	110.88(8), 110.88(8)			–	269 (H-C)
LEZQIQ	1999	C_6H_4-4-NO_2	C_6H_4-4-NO_2	–				291.4(3) (O)	266 (H-C)

4. Computational Studies

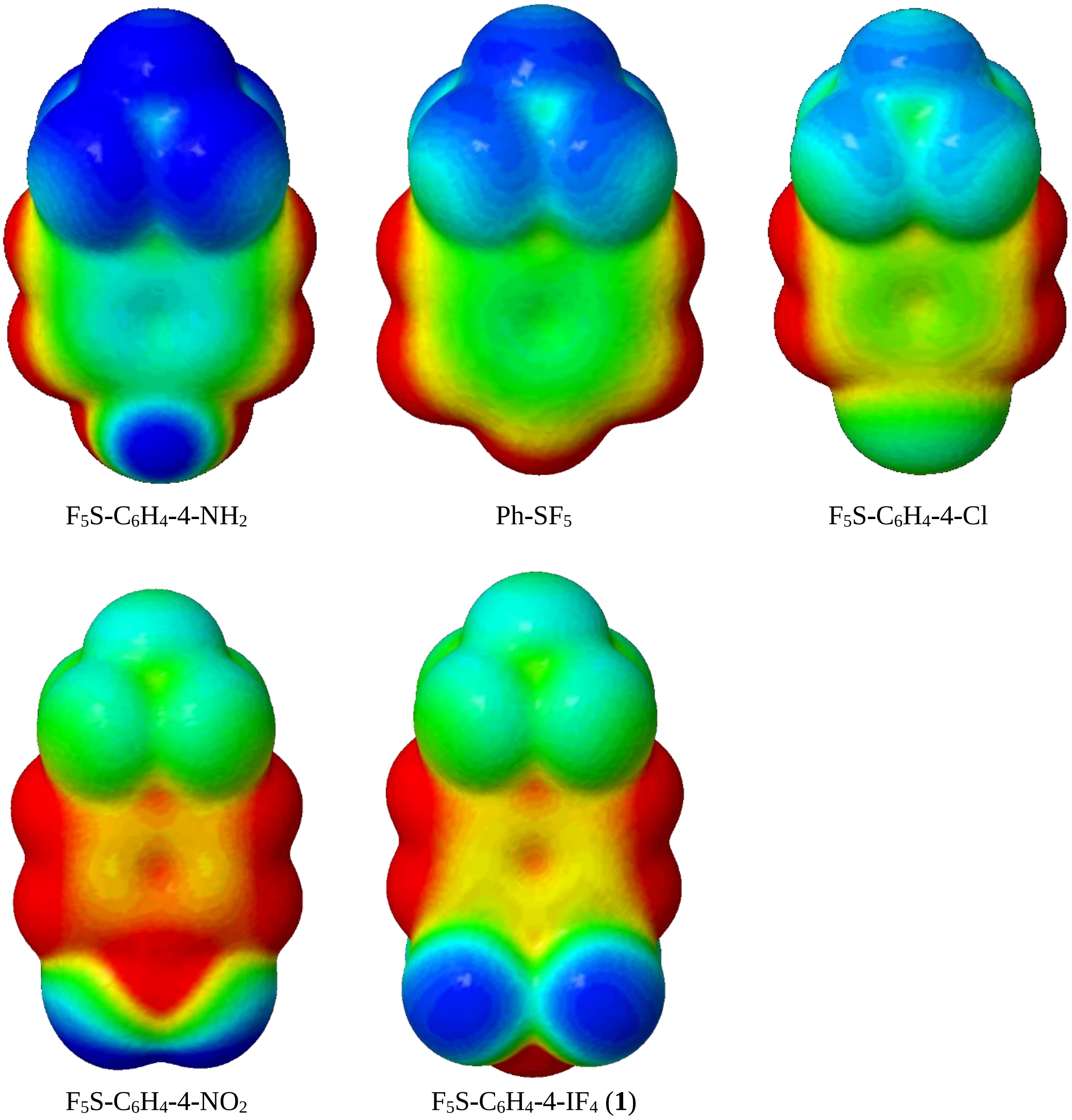

Figure S15. Representation of the electrostatic potential surface of selected SF_5-substituted molecules, calculated at the SCS-MP2/aug-cc-pVTZ level of theory and shown on a density iso-value surface of 0.001 a.u. (colour scale from –15 kcal/mol, blue; to +15 kcal/mol, red).

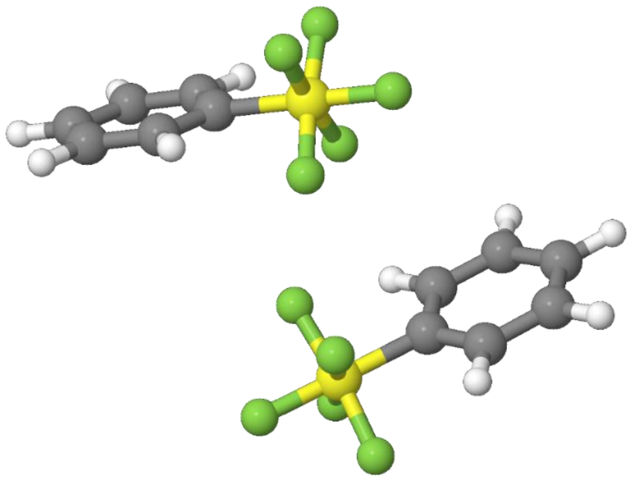

d(F···F) = 269.3 pm, ΔE_{int} = –10.1 kJ/mol

Figure S16. Partially optimised structure of a $(Ph\text{-}SF_5)_2$ dimer for interaction-energy estimates, formed by an $F_{eq}\cdots F_{eq}$ contact (motif B in Scheme 2 in the main text; *anti* isomer). The motif was extracted from the crystal structure dataset HOMSOT, the substituents replaced by H atoms, and the SF_5 groups were fixed during optimisation. The interaction energy was estimated at the SCS-MP2-F12/cc-pVTZ-F12 (CP corrected) level of theory.

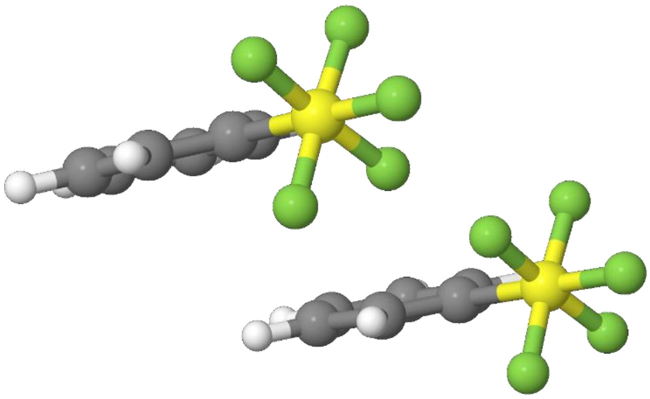

d(F···F) = 258.8 pm, ΔE_{int} = –9.3 kJ/mol

Figure S17. Partially optimised structure of a $(Ph\text{-}SF_5)_2$ dimer for interaction-energy estimates, formed by an $F_{eq}\cdots F_{eq}$ contact (motif B in Scheme 2 in the main text; *syn* isomer). The motif was extracted from the crystal structure dataset GEFZIC01, the substituents replaced by H atoms, and the SF_5 groups were fixed during optimisation. The interaction energy was estimated at the SCS-MP2-F12/cc-pVTZ-F12 (CP corrected) level of theory.

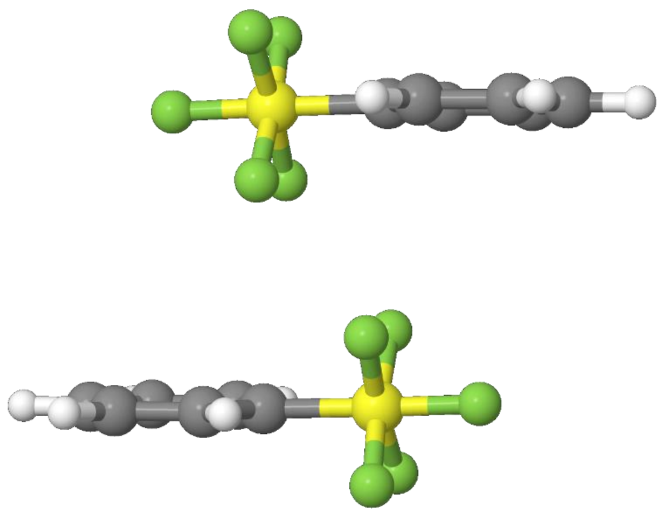

d(F···F) = 289.4 pm, ΔE_{int} = –13.5 kJ/mol

Figure S18. Partially optimised structure of a $(Ph\text{-}SF_5)_2$ dimer for interaction-energy estimates, formed by a dual $F_{eq}\cdots F_{eq}$ contact (motif C in Scheme 2 in the main text). The motif was extracted from the crystal structure dataset OHOGOJ, the substituents replaced by H atoms, and the SF_5 groups were fixed during optimisation. The interaction energy was estimated at the SCS-MP2-F12/cc-pVTZ-F12 (CP corrected) level of theory.

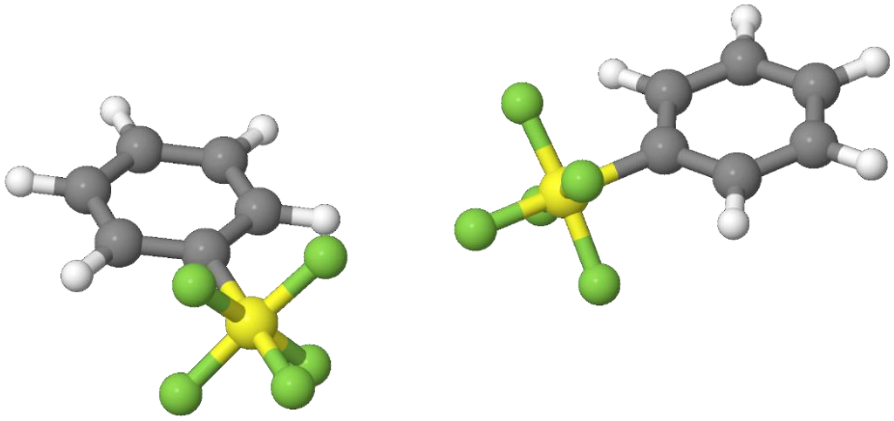

d(F···F) = 272.1 pm, ΔE_{int} = –4.6 kJ/mol

Figure S19. Partially optimised structure of a $(Ph\text{-}SF_5)_2$ dimer for interaction-energy estimates, formed by an $F_{eq}\cdots F_{ax}$ contact (motif E in Scheme 2 in the main text). The motif was extracted from the crystal structure dataset DAPLIS, the substituents replaced by H atoms, and the SF_5 groups were fixed during optimisation. The interaction energy was estimated at the SCS-MP2-F12/cc-pVTZ-F12 (CP corrected) level of theory.

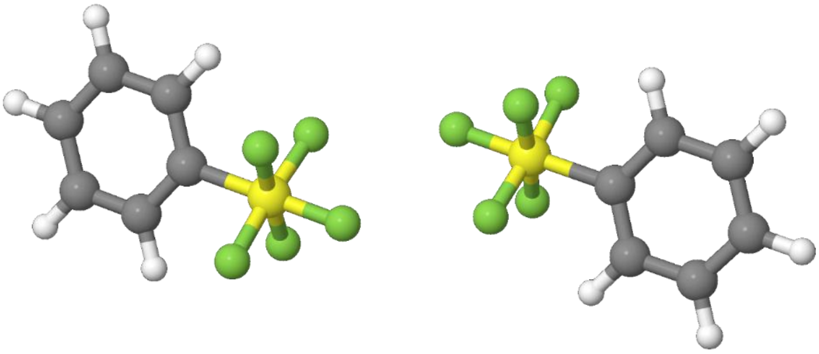

d(F···F) = 293.2 pm, ΔE_{int} = –2.1 kJ/mol

Figure S20. Partially optimised structure of a $(Ph\text{-}SF_5)_2$ dimer for interaction-energy estimates, formed by a dual $F_{eq}\cdots F_{ax}$ contact (motif F in Scheme 2 in the main text). The motif was extracted from the crystal structure dataset QAFGOT, the substituents replaced by H atoms, and the SF_5 groups were fixed during optimisation. The interaction energy was estimated at the SCS-MP2-F12/cc-pVTZ-F12 (CP corrected) level of theory.

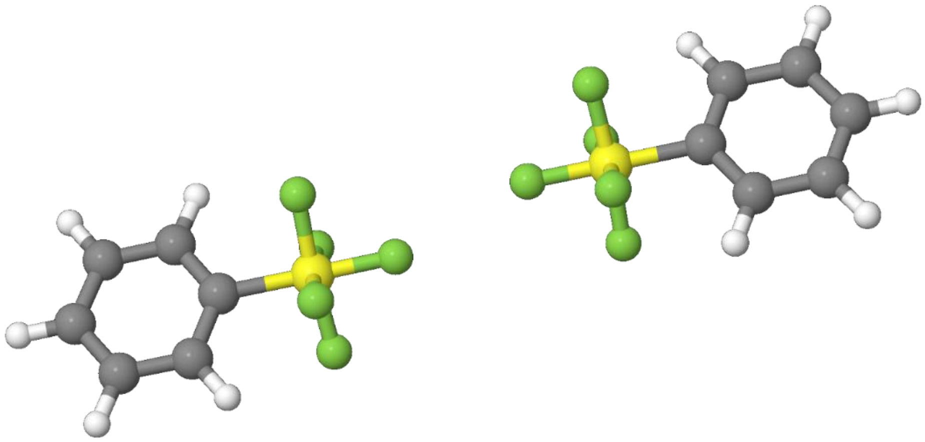

d(F···F) = 285.8 pm, ΔE_{int} = –0.1 kJ/mol

Figure S21. Partially optimised structure of a $(Ph\text{-}SF_5)_2$ dimer for interaction-energy estimates, formed by an $F_{eq}\cdots F_{ax}$ contact (motif G in Scheme 2 in the main text). The motif was extracted from the crystal structure dataset IWUFUD, the substituents replaced by H atoms, and the SF_5 groups were fixed during optimisation. The interaction energy was estimated at the SCS-MP2-F12/cc-pVTZ-F12 (CP corrected) level of theory.

References

[S1] M. J. Turner, J. J. McKinnon, S. K. Wolff, D. J. Grimwood, P. R. Spackman, D. Jayatilaka, M. A. Spackman, *CrystalExplorer17* (2017). University of Western Australia. https://hirshfeldsurface.net.

[S2] URL: https://crystalexplorer.scb.uwa.edu.au/wiki/index.php/Fingerprint_Plots.
